# α-Glycosidase activity of novel coumarin–triazole–coumarin dyads

**DOI:** 10.55730/1300-0527.3770

**Published:** 2025-10-27

**Authors:** Ersin ŞİRİN, Esra SEVİMLİ, Gökçe SEYHAN, Burak BARUT, Yunus KAYA, Baybars KÖKSOY

**Affiliations:** 1 Department of Chemistry, Faculty of Engineering and Natural Sciences, Bursa Technical University, Bursa, Turkiye; 2 Department of Biochemistry, Faculty of Pharmacy, Karadeniz Technical University, Trabzon, Turkiye

**Keywords:** Coumarin, triazole, enzyme inhibition, α-glycosidase

## Abstract

Novel coumarin–triazole–coumarin dyads were synthesized and characterized, and their α-glycosidase inhibitory activities were evaluated spectrophotometrically. Compound 4e exhibited the most pronounced inhibitory effect, with an IC_50_ value of 38.98 ± 0.77 μM. The IC_50_ values for 4d and 4a were 93.55 ± 1.70 μM and 95.04 ± 3.55 μM, respectively. The Lineweaver–Burk plot showed that 4e inhibited α-glycosidase in a mixed type. In addition, the *K_i_* value obtained from the Dixon plot was 19.95 ± 0.15 μM for α-glycosidase.

## 1. Introduction

α-Glycosidase, a membrane-bound enzyme located in the brush border of the small intestinal epithelium, catalyzes the final step in the carbohydrate digestion process by hydrolyzing oligosaccharides and disaccharides into absorbable monosaccharides, primarily glucose [1,2]. This enzymatic activity plays a critical role in the regulation of postprandial blood glucose levels. Therefore, inhibition of α-glycosidase has emerged as an effective therapeutic strategy for controlling postprandial hyperglycemia and managing Type II Diabetes Mellitus [3,4]. Clinically, α-glycosidase inhibitors such as acarbose, miglitol, and voglibose are widely used to delay carbohydrate digestion and glucose absorption, thereby reducing postprandial blood glucose spikes [5]. However, the long-term use of these synthetic inhibitors is often associated with undesirable side effects, including flatulence, diarrhea, abdominal discomfort, and in some cases, hepatic complications [6,7]. These adverse effects highlight the need for novel α-glycosidase inhibitors with greater therapeutic efficacy, improved safety profiles, and minimal side effects.

Coumarin, also known as 2*H*-chromen-2-one, contains a fused benzene and α-pyrone ring. Coumarin derivatives are widely distributed in nature and can be found in plants, fungi, and bacteria. They are present in various plant parts, including fruits, roots, seeds, and leaves [8–10]. The natural occurrence of this class of compounds provides a significant advantage for researchers, particularly in terms of low toxicity during biological activity studies. Numerous studies have reported that coumarins exhibit diverse pharmacological properties, including anticancer [11], antiinflammatory [12], antitumor [13], and antihyperlipidemic [14] activities. Due to their structural flexibility, favorable physicochemical properties, and ability to interact with multiple biological targets, coumarins are regarded as privileged scaffolds in medicinal chemistry. Their low molecular weight, high bioavailability, and low toxicity make them valuable lead compounds in drug discovery [15]. Moreover, recent studies have demonstrated that the hybridization of coumarin with nitrogen-containing heterocycles, particularly triazole rings, which can be efficiently linked via click chemistry [16–19], can synergistically enhance biological activity. Such coumarin-triazole hybrids exhibit improved pharmacokinetic properties and enzyme-binding affinities, primarily through electronic and steric interactions between the two moieties, confirming their potential as multifunctional pharmacophores [20]. Following the pharmacological significance of coumarin, the triazole ring itself represents another highly versatile heterocyclic motif widely recognized in medicinal chemistry. Triazoles (also referred to as pyrrodiazoles) are five-membered unsaturated ring systems containing three nitrogen atoms within the heterocyclic core [21,22]. 1,2,3-Triazole groups are known for their exceptional chemical stability toward hydrolysis, oxidation, reduction, and enzymatic degradation. Moreover, their ability to form hydrogen bonds, dipole-dipole, and π-π stacking interactions enhances their affinity for biological targets [23,24]. Triazole derivatives have been shown to exhibit a broad spectrum of biological activities, including anticancer [25], antioxidant [26], antifungal [27], and antiinflammatory [28] effects. Despite growing interest in coumarin-triazole chemistry, their potential as α-glycosidase inhibitors relevant to diabetes therapy has not been systematically investigated. Recent findings indicate that these hybrids may serve as promising enzyme inhibitors; however, further studies are required to clarify their structure–activity relationships and molecular inhibitory mechanisms.

In this context, the present study was designed to synthesize and characterize five novel triazole-coumarin hybrids that integrate two pharmacologically privileged scaffolds within a single molecular framework. The α-glycosidase inhibitory activities of the synthesized compounds were examined using in vitro assays and molecular docking to elucidate the relationship between molecular structure and inhibitory efficiency against α-glycosidase. The combined kinetic and computational analyses not only highlight the potential of these hybrids as promising antidiabetic candidates but also provide mechanistic insights that may guide the rational design of next-generation α-glycosidase inhibitors.

## 2. Materials and methods

### 2.1. Materials

**Figure 1:**
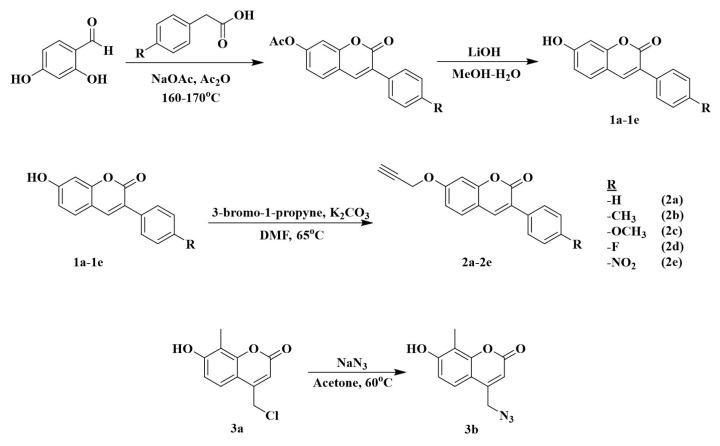
Synthetic route of starting materials.

**Figure 2:**
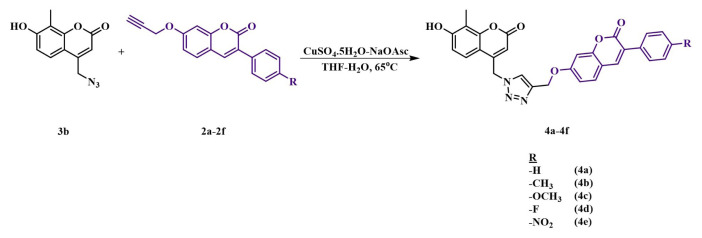
Synthetic route of target coumarin–triazole–coumarin derivatives.

The following chemicals were purchased from Sigma-Aldrich: phenylacetic acid (99%), sodium acetate (≥ 99.9%), sulfuric acid (≥ 99.9%), sodium azide (≥ 99.9%), acetone (≥ 99.9%), methanol (≥ 99.9%), lithium hydroxide (98%), hydrochloric acid (37%), propargyl bromide (80%), dimethyl formamide (DMF) (≥ 99.9%), tetrahydrofuran (THF) (≥ 99.9%), potassium carbonate (≥ 99.9%), sodium ascorbate (NaOAsc) (≥ 98%), copper sulfate pentahydrate (≥ 98%), chloroform (≥ 99.9%), dichloromethane (≥ 99.8%), diethyl ether (≥ 99.9%), agarose, acetic acid (≥ 99%), acetylcholinesterase, acetylthiocholine iodide (≥ 98%), butyrylcholinesterase, butyrylthiocholine iodide (≥ 99%), 5,5’-dithiobis(2-nitrobenzoic acid) (≥ 98%), 2,2-diphenyl-1-picrylhydrazyl, ethidium bromide, α-glycosidase enzyme, hydrogen peroxide (30-31%), glycerol (≥ 99%), 4-nitrophenyl-α-D-glucopyranoside, sodium dodecyl sulfate (SDS) (≥ 98.5%), trizma base (≥ 99.9%); Tokyo Chemical Industry: 2-methylresorcinol (> 98%), ethyl 4-chloroacetoacetate (> 95%), 2,4-dihydroxybenzaldehyde (> 98%), *p*-tolylacetic acid (> 98%), 4-methoxyphenylacetic acid (> 98%), 4-nitrophenylacetic acid (> 98%), 4-fluorophenylacetic acid (> 98%); Thermo Scientific: pBR322 plasmid DNA. The purity of the products was controlled by thin-layer chromatography (silica gel F-254-coated thin-layer chromatography plate). The novel triazole–coumarin compounds were characterized by Fourier transform infrared (FT-IR) spectroscopy, ^1^H and ^13^C nuclear magnetic resonance (NMR) spectroscopy, and mass spectrometry. A PerkinElmer Spectrum Two FT-IR Spectrometer was used for FT-IR spectra, a Bruker Microflex LT MALDI-TOF (Matrix-Assisted Laser Desorption/Ionization–Time-of-Flight) was used for mass spectra, and a JEOL ECX-400 MHz and a Bruker DPX-400 High-Performance FT-NMR were used to record ^1^H and ^13^C NMR spectra with tetramethylsilane (TMS) as an internal standard. Enzyme inhibition and kinetic analyses were performed using a Thermo Scientific MultiskanTM GO Microplate reader. The synthesis of the starting materials is presented in Figure 1 (also provided as Figure S1 in the Supporting information), and the synthesis of the target compounds is shown in Figure 2.

### 2.2. General synthesis of compounds

#### 2.2.1. Synthesis of triazole–coumarins

Azide–coumarin derivative 3b (0.86 mmol, 200 mg) and 0,95 mmol 2a–2f (262.48 mg 2a, 275.8 mg 2b, 291 mg 2c, 279.56 mg 2d, 305.23 mg 2e) were dissolved in 7 mL THF and 0.5 mL DMF. After that, CuSO_4_·5H_2_O (0.86 mmol, 214.73 mg) and NaOAsc (0.52 mmol, 103 mg) were dissolved in distilled water (0.111 mol, 2 mL) and slowly added to the reaction medium. The reaction mixture was stirred for 24 h at 60–65 °C. After completion, the reaction was cooled and poured into ice water. The precipitate (4a–4e) was filtered and washed with water, under argon atmosphere, methanol, dichloromethane, chloroform, and diethyl ether, respectively.

##### 2.2.1.1. 7-Hydroxy-8-methyl-4-((4-(((2-oxo-3-phenyl-2*H*-chromen-7-yl)oxy)methyl)-1*H*-1,2,3-triazol-1-yl)methyl)-2*H*-chromen-2-one (4a)

Light brown solid, yield: 70.4%, m.p. 200 °C (decomposed). FTIR [ATR], cm^−1^: 3564–3200 (ʋOH), 3088 (ʋArC-H), 2970 (ʋAlC-H), 1713 (ʋC=O), 1606 (ʋC=C), 1581 (triazole ʋN=N). ^1^H NMR (400 MHz, deuterated dimethyl sulfoxide [DMSO]-d6) δ (ppm): 10.62 (s, 1H), 8.45 (s, 1H), 8.22 (s, 1H), 7.74 (d, *J* = 1.7 Hz, 1H), 7.72 (dd, *J* = 5.3, 3.5 Hz, 2H), 7.56 (d, *J* = 8.7 Hz, 1H), 7.50–7.43 (m, 2H), 7.44–7.38 (m, 1H), 7.22 (d, *J* = 2.4 Hz, 1H), 7.07 (dd, *J* = 8.6, 2.4 Hz, 1H), 6.89 (d, *J* = 8.7 Hz, 1H), 5.94 (s, 2H), 5.61 (s, 1H), 5.34 (s, 2H), 2.17 (s, 3H). ^13^C NMR (100 MHz, DMSO-d6) δC (ppm): 161.4, 160.5, 160.4, 159.9, 155.2, 153.5, 151.1, 143.0, 141.2, 135.4, 130.2, 128.8, 128.6, 126.5, 123.9, 123.1, 113.8, 113.7, 112.5, 111.7, 109.8, 109.5, 101.6, 62.1, 49.8, 8.5. MALDI–TOF MS (m/z): 507.599 [M]^+^ calculated for C_29_H_21_N_3_O_6_; 507.14, CHN Analysis. Calculated for C_29_H_21_N_3_O_6_ (507.14 g/mol): C, 68.63; H, 4.17; N, 8.28; Found C, 68.61; H, 4.14; N, 8.26.

##### 2.2.1.2. 7-Hydroxy-8-methyl-4-((4-(((2-oxo-3-(p-tolyl)-2*H*-chromen-7-yl)oxy)methyl)-1*H*-1,2,3-triazol-1-yl)methyl)-2*H*-chromen-2-one (4b)

Cream solid, yield: 71.2%, m.p. 260 °C (decomposed). FTIR (ATR, cm^−1^): 3426–3200 (ʋOH), 3097 (ʋArC-H), 2921 (ʋAlC-H), 1728 (ʋC=O), 1608 (ʋC=C), 1582 (triazole ʋN=N). ^1^H NMR (400 MHz, DMSO-d6) δ (ppm): 10.56 (s, 1H), 8.44 (s, 1H), 8.17 (s, 1H), 7.70 (d, *J* = 8.6 Hz, 1H), 7.63 (d, *J* = 7.8 Hz, 2H), 7.56 (d, *J* = 8.6 Hz, 1H), 7.27 (d, *J* = 7.7 Hz, 2H), 7.19 (s, 1H), 7.06 (d, *J* = 8.8 Hz, 1H), 6.89 (d, *J* = 8.7 Hz, 1H), 5.93 (s, 2H), 5.63 (s, 1H), 5.34 (s, 2H), 2.36 (s, 3H), 2.17 (s, 3H). ^13^C NMR (100 MHz, DMSO-d6) δC (ppm): 161.3, 160.5, 160.5, 159.9, 155.0, 153.5, 151.1, 143.0, 140.6, 138.7, 132.5, 130.1, 129.3, 128.6, 126.5, 123.8, 123.1, 113.8, 113.6, 112.5, 111.6, 109.8, 109.5, 101.6, 62.1, 49.8, 21.3, 8.5. MALDI–TOF MS (m/z): 544,361 [M+Na]^+^, 521.256 [M]^+^ calculated for C_30_H_23_N_3_O_6_; 521.16, CHN Analysis. Calculated for C_30_H_23_N_3_O_6_ (521.16 g/mol): C, 69.09; H, 4.45; N, 8.06; found C, 69.06; H, 4.42; N, 8.04.

##### 2.2.1.3.7-Hydroxy-4-((4-(((3-(4-methoxyphenyl)-2-oxo-2*H*-chromen-7-yl)oxy)methyl)-1*H*-1,2,3-triazol-1-yl)methyl)-8-methyl-2*H*-chromen-2-one (4c)

Light brown solid, yield: 70.3%, m.p. 211 °C (decomposed). FTIR (ATR, cm^−1^): 3252 (ʋOH), 3080 (ʋArC-H), 2969 (ʋAlC-H), 1711 (ʋC=O), 1608 (ʋC=C), 1580 (triazole ʋN=N^1^H NMR). (400 MHz, DMSO-d6) δ (ppm): 10.67 (s, 1H), 8.45 (s, 1H), 8.15 (s, 1H), 7.75–7.69 (m, 2H), 7.69–7.65 (m, 1H), 7.59–7.53 (m, 1H), 7.20 (s, 1H), 7.05 (d, *J* = 12.4 Hz, 2H), 7.01 (s, 1H), 6.89 (d, *J* = 8.6 Hz, 1H), 5.94 (s, 2H), 5.61 (s, 1H), 5.33 (s, 2H), 3.82 (s, 3H), 2.17 (s, 3H). ^13^C NMR (100 MHz, DMSO-d6) δC (ppm): 161.1, 160.6, 160.5, 159.9, 159.8, 154.8, 153.5, 151.1, 143.0, 139.8, 130.1, 127.6, 126.5, 123.6, 123.1, 114.1, 114.0, 113.6, 112.5, 111.6, 109.8, 109.5, 101.6, 62.1, 55.7, 49.8, 8.5. MALDI–TOF MS (m/z): 537.876 [M]^+^ calculated for C_30_H_23_N_3_O_7_; 537.53, CHN analysis. Calculated for C_30_H_23_N_3_O_7_ (537.53 g/mol): C, 67.03; H, 4.31; N, 7.82; Found C, 67.02; H, 4.31; N, 7.81.

##### 2.2.1.4. 4-((4-(((3-(4-Fluorophenyl)-2-oxo-2*H*-chromen-7-yl)oxy)methyl)-1*H*-1,2,3-triazol-1-yl)methyl)-7-hydroxy-8-methyl-2*H*-chromen-2-one (4d)

Dark yellow solid, yield: 69.9%, m.p. 210 °C (decomposed). FTIR (ATR, cm^−1^): 3227 (O-H), 3074 (ʋArC-H), 2923 (ʋAlC-H), 1716 (ʋC=O), 1606 (ʋC=C), 1513 (triazole ʋN=N). ^1^H NMR (400 MHz, DMSO-d6) δ (ppm): 10.63 (s, 1H), 8.45 (s, 1H), 8.22 (s, 1H), 7.78 (dd, *J* = 8.5, 5.5 Hz, 2H), 7.71 (d, *J* = 8.7 Hz, 1H), 7.56 (d, *J* = 8.7 Hz, 1H), 7.30 (t, *J* = 8.8 Hz, 2H), 7.22 (d, *J* = 2.3 Hz, 1H), 7.08 (d, *J* = 8.6 Hz, 1H), 6.89 (d, *J* = 8.8 Hz, 1H), 5.94 (s, 2H), 5.60 (s, 1H), 5.34 (s, 2H), 2.17 (s, 3H). ^13^C NMR (100 MHz, DMSO-d6) δC (ppm): 163.7, 161.4, 161.2, 160.5, 160.4, 159.9, 155.2, 153.5, 151.1, 141.2, 131.8, 131.0, 130.2, 123.1, 122.9, 115.7, 115.5, 113.8, 113.7, 112.5, 111.7, 109.8, 109.5, 101.7, 62.1, 49.8, 8.5. MALDI–TOF MS (m/z): 525.754 [M]^+^ calculated for C_29_H_20_FN_3_O_6_; 525.49, CHN Analysis. Calculated for C_29_H_20_FN_3_O_6_ (525.49 g/mol): C, 66.28; H, 3.84; N, 8.00; Found C, 66.25; H, 3.83; N, 7.98.

##### 2.2.1.5. 7-Hydroxy-8-methyl-4-((4-(((3-(4-nitrophenyl)-2-oxo-2*H*-chromen-7-yl)oxy)methyl)-1*H*-1,2,3-triazol-1-yl)methyl)-2*H*-chromen-2-one (4e)

Brown solid, yield: 70.1%, m.p. 205 °C (decomposed). FTIR (ATR, cm^−1^): 3574–3201 (ʋOH), 3073 (ʋArC-H), 2932 (ʋAlC-H), 1712 (ʋC=O), 1607 (ʋC=C), 1580 (triazole ʋN=N). ^1^H NMR (400 MHz, DMSO-d6) δ (ppm): 8.40 (s, 2H), 8.28 (d, *J* = 9.0 Hz, 2H), 8.03–7.96 (m, 2H), 7.71 (d, *J* = 8.7 Hz, 1H), 7.43 (d, *J* = 8.8 Hz, 1H), 7.22 (d, *J* = 2.5 Hz, 1H), 7.06 (dd, *J* = 8.9, 2.6 Hz, 1H), 6.74 (d, *J* = 8.6 Hz, 1H), 5.86 (s, 2H), 5.38 (s, 1H), 5.31 (s, 2H), 2.08 (s, 3H). ^13^C NMR (100 MHz, DMSO-d6) δC (ppm): 162.1, 160.5, 159.9, 155.6, 153.5, 151.1, 147.3, 143.3, 142.1, 130.8, 129.9, 126.5, 125.5, 123.8, 123.1, 121.7, 114.0, 113.6, 112.4, 111.7, 109.8, 109.5, 101.7, 62.2, 49.8, 29.5, 8.5. MALDI–TOF MS (m/z): 552.501 [M]^+^ calculated for C_29_H_20_N_4_O_8_; 552.50, CHN Analysis. Calculated for C_29_H_20_N_4_O_8_ (552.50 g/mol): C, 63.04; H, 3.65; N, 10.14; Found C, 63.03; H, 3.64; N, 10.12.

#### 2.2.2. α-Glycosidase inhibitory assay

The α-glycosidase inhibitory effects of the compounds were determined as described in the previous study [29]. Acarbose (Sigma-Aldrich, A8980) was used as a reference compound in this study. The compounds were first dissolved in 25 mM DMSO and then diluted to 1 mM with phosphate buffer (0.1 M, pH: 6.9). In brief, the compounds and acarbose in phosphate buffer (0.1 M, pH: 6.9) were added to a 96-well plate at 5–250 μM. Then, 100 μL, 0.5 U/mL α-glycosidase (Sigma-Aldrich, G5003) was added to the wells. After 10 min, 50 μL of 5 mM p-nitrophenyl-α-D-glucopyranoside (4-pNPG) (Sigma-Aldrich, 487506) was added as a substrate to each well. After incubation for 10 min, spectrophotometric measurement was performed at 405 nm. The percentage inhibition rate of the compounds was calculated using the following formula:


$$Inhibition\%=(A_{0}-A_{1}/A_{0})\times 100$$

In this formula, A_0_ and A_1_ represent the absorbance of the mixture in the absence and presence of the compound, respectively. In addition, IC50 values, which indicate the enzyme concentration required to inhibit half of the enzyme activity, were calculated.

Following determination of the α-glycosidase inhibitory effects of the compounds, kinetic analyses were performed to determine the mode of inhibition for 4e, which showed the greatest impact. To determine this, the final concentration of α-glycosidase was fixed at 0.5 U/mL. Then, different concentrations of 4-pNPG (2.5–20 mM) and varying concentrations of 4e (20 μM, 40 μM, and 100 μM) showing inhibitory potential were added to the wells. Following incubation, the absorbance values at 405 nm in the presence and absence of the compound were measured in the microplate reader. Lineweaver–Burk and Dixon plots were used to determine the inhibitor type and the inhibitory constant (*K_i_*) of 4e [30,31]. In the Lineweaver–Burk plot, the *y*-axis represents 1/*V*, the inverse of the reaction rate, while the *x*-axis represents 1/[S], the inverse of the substrate concentration. According to this plot, the points intersecting the *y*-axis give the value 1/*V_max_*. From here, the *V_max_* value, which represents the maximum reaction rate, can be determined. *K_m_*, a value indicating the enzyme’s interest in the substrate, can be determined from the points where the line intersects the *x*-axis. The type of inhibition is then determined by analyzing the changes in *V_max_* and *K_m_*. The Dixon graph is used to calculate the *K_i_* value, which indicates the inhibitory potential of 4e [32].

### 2.3. Theoretical studies

Optimization and frequency calculations of coumarin–triazole–coumarin dyads derivative compounds (4a–4e) were performed by using the Gaussian 09 program. GaussView 5.0 software was used to visualize the results and to create input files [33]. First of all, the optimization of all molecules was carried out by density functional theory (DFT/B3LYP) with the 6–311++G(d,p) basis set [34]. Frequency calculations were performed using the same method and basis set to verify that the structure is at the actual minimum on the potential energy surface. So, the most probable molecular geometries were determined. Later, all molecules (4a–4e) used in the molecular docking studies were converted to .mol2 files using GaussView 5.0. a-Glycosidase enzymes (PDB ID: 3A4A) were retrieved from the RCSB^1^ Protein Data Bank. AutoDock Tool 4.2 was used to prepare all molecules and proteins for molecular docking studies. Polar hydrogen atoms were added to the receptor structure of the proteins. In addition, the proteins were prepared for docking by removing unwanted molecules and water. Docking of all molecules was carried out with AutoDockVina [35]. The other parameters used in the calculations are data determined by the program itself. After the docking study was completed, the best conformation with the lowest docked energy was selected. Protein–ligand conformations, including hydrogen bonds, π-π, and hydrophobic interactions, were analyzed using the Discovery Studio 3.5 client [36].

For in silico studies, ADME screening and drug similarity assessment were performed using the web tool SwissADME^2^ developed by the Swiss Bioinformatics Institute [37]. In the study, compounds with high binding energy, which are potential drug candidates, were subjected to this part of the screening process. Some simple physicochemical properties, Molecular weight (MW), molecular fracture (MR), atomic number, and polar surface area (PSA) were calculated within the scope of the study. Drug similarity candidacy was applied by Lipinski [38], Ghose [39], and Veber [40] using the rule of 5 (RO5) screening. The solubility (log S) of the selected ligands was determined by applying the ESOL model [41].

### 2.4. Statistical analysis

GraphPad Prism Software version 5.0 (San Diego, California, USA) was used for all the statistical analyses. Statistical analysis was performed by one-way analysis of variance (ANOVA) followed by Tukey’s tests for enzyme inhibition.

## 3. Result and discussion

### 3.1. Chemistry

Five new coumarin–triazole derivatives (4a, 4e) were synthesized by the click reaction of 4-(azidomethyl)-7-hydroxy-8-methyl-2H-chromen-2-one (3b) and compounds 2a, 2e in the presence of CuSO_4_.5H_2_O and NaOAsc (Figure 2). After purification of the synthesized derivatives, structural characterization was performed using standard spectroscopic methods, including FTIR, ^1^H NMR, ^13^C NMR, and mass spectrometry (Figures S2–S6).

As the IR spectra of the synthesized derivatives (4a–4e) were examined, the broad bands of the ʋ(-OH) group present in the coumarin ring were observed in the range of 3574–3200 cm^−1^. The ʋ(N_3_) band of the starting compound azido coumarin (3b) appears at 2108 cm^−1^, while the alkyne coumarin intermediates (2a–2e) exhibited terminal alkyne ʋ(C≡C–H) at 3291–3251 cm^−1^ and ʋ(C≡C) at 2129–2121 cm^−1^. In the products (4a–4e), the disappearance of the azide (≈2108 cm^−1^) and alkyne (≈2129–2121; 3291–3251 cm^−1^) stretching bands, consistent with the literature, evidences triazole formation and confirms the successful synthesis of the target molecules [42]. The ʋ(ArC-H) bands of compounds 4a–4e are in the range 3097–3074 cm^−1^, ʋ(AlC-H) bands 2970–2921 cm^−1^, ʋ(C=O) 1728–1711 cm^−1^, and ʋ(C=C) bands 1608–1606 cm^−1^.

In the ^1^H NMR spectra of the synthesized compounds (4a–4e), the chemical shift values were observed in the range of δ 10.67–2.08 ppm. It is seen that the peaks belonging to the phenolic -OH group of derivatives 4a–4e occur in the range of δ 10.67–10.54 ppm, and the aromatic peaks appear in the range of δ 8.28–6.54 ppm. The -C=C-H peaks in the 3rd position of coumarin, which are among the characteristic peaks of coumarin groups, were observed at δ 5.86–5.60 ppm, while the -C=C-H peaks in the 4th position, which is the other characteristic peak, were observed in the range of δ 8.45–8.40 ppm [43,44]. The -C=C-H peaks of the triazole group of compounds 4a–4e occurred in the range of δ 8.40–8.15 ppm [45,46]. The ^1^H NMR spectra of the synthesized compounds exhibited characteristic coumarin and triazole proton resonances within the expected chemical shift ranges. These data are in good agreement with previously reported values for coumarin– and triazole-containing systems [43,44].

As the MALDI–TOF mass spectra of compounds 4a–4e were analyzed, it was observed that while the calculated molecular weight of compound 4b was 521.53 g/mol, the [M^+^Na]^+^ peak was 544.361 g/mol and the [M]^+^ peak was 521.256 g/mol as a result of the compound capturing a Na atom. The computed molecular weight values of the remaining compounds (4a, 4c–4e) have been found to be consistent with the experimental [M]^+^ value. Interestingly, among the synthesized coumarin–triazole–coumarin derivatives, compound 4b (bearing a para-CH_3_ substituent) exhibited both [M]^+^ and [M^+^Na]^+^ peaks in its mass spectrum, whereas the other compounds primarily showed only [M]^+^ ions. Although the -OCH_3_ group in compound 4c is generally considered a stronger electron-donating substituent than -CH_3_, the appearance of the sodium adduct in 4b is thought to be influenced not only by electronic effects but also by factors such as steric accessibility, local charge distribution, and the ionization environment during mass spectrometric analysis. These structural and polarity-related factors may facilitate sodium ion coordination in 4b, leading to the observed [M^+^Na]^+^ species.

### 3.2. α-Glycosidase inhibitory effects of the compounds

The inhibitory effects of the compounds on α-glycosidase were examined, and the results are presented in Table 1. The results of the experiment indicated that among the studied compounds, 4e exhibited the most pronounced inhibitory effect, with an IC_50_ value of 38.98 ± 0.77 μM (R^2^ = 0.98; y = 0.6523x + 24.573). The IC_50_ values of 4d and 4a were calculated as 93.55 ± 1.70 μM (R^2^ = 0.9599; y = 0.5178x + 1.5577) and 95.04 ± 3.55 μM (R^2^ = 0.9969; y = 0.4865x–3.7619), respectively. On the other hand, the IC_50_ values for 4b and 4c were 122.57 ± 4.20 μM and 108.97 ± 2.40 μM, respectively. These values revealed that these compounds had weak effects on α-glycosidase. Furthermore, a comparison between 4e and acarbose (IC_50_ = 64.49 ± 2.53 μM) showed that 4e was more effective against α-glycosidase. In the presence of 4a, there is -H instead of the R group, and the IC_50_ value of this compound was found to be 95.04 ± 3.55 μM. In the presence of 4e, it was determined that the IC_50_ value decreased to 38.98 ± 0.77 μM with the addition of -NO_2_ instead of the R group. This situation revealed that the activity against glycosidase increased with the addition of the nitro group to the compound. This result was also consistent with the literature [47]. Triazole/coumarin compounds are expected to exhibit stronger inhibitory effects due to stronger interactions with the target via hydrogen bonds.

**Figure 3:**
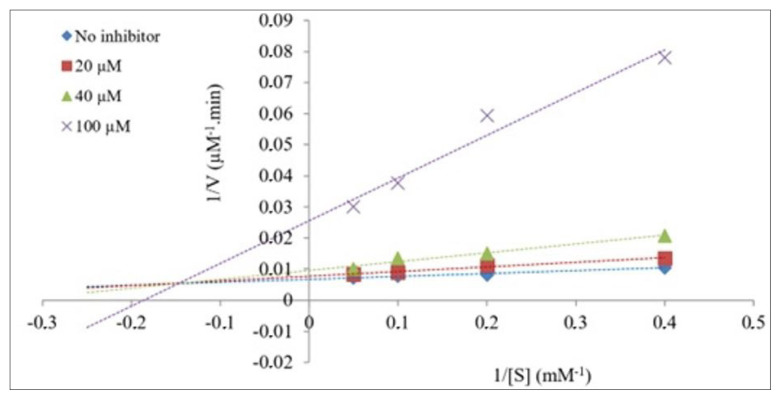
Lineweaver–Burk plot of 4e on α-glycosidase.

**Figure 4:**
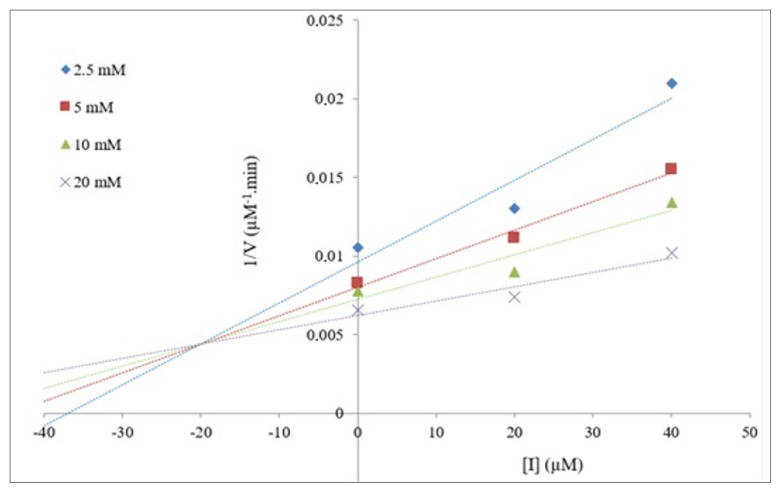
Dixon plot of 4e on α-glycosidase.

The results of kinetic studies with compound 4e are given in Table 1. The changes in *V_max_* (decrease) and *K_m_* (increase) values obtained from the Lineweaver–Burk plot indicated that 4e inhibited α-glycosidase in a mixed-type manner (Figure 3). In addition, the *K_i_* value obtained from the Dixon plot was 19.95 ± 0.15 μM for α-glycosidase (Figure 4).

### 3.3. Optimized studies

Optimized structures of molecules obtained by DFT calculations (shown in Figure S7), some physicochemical properties such as highest occupied molecular orbital (HOMO), lowest unoccupied molecular orbital (LUMO) energy, band gap, dipole moment, and polarization, and the relationship between these calculations and inhibition efficiency are discussed in the supporting file.

### 3.4. Molecular docking studies

All synthesized compounds showed inhibitory activity against the α-glycosidase enzyme. Therefore, all the synthesized compounds were placed in the binding cavity of α-glycosidase. The best binding affinity of each compound and types of receptor–ligand interactions were evaluated, and the well-known interactions of the compounds within the active pocket of receptor target proteins are listed in Table 2. Molecular docking calculations were performed using the crystal structure of α-glycosidase with PDB ID: 3A4A, which corresponds to the isomaltase enzyme derived from Saccharomyces cerevisiae. This yeast-derived enzyme is a well-characterized model of α-glycosidase activity and is frequently used in computational studies due to its clearly defined active-site architecture. RMSD values were calculated for the input geometries used in docking calculations for all molecules. If the RMSD is less than 2 Å, the docking process is considered successful [48]. Visual and RMSD inspection of the binding site and known binding poses confirmed that the docking scoring function was able to select the correct pose for these molecules. When the mode analyses of all molecules were performed, it was determined that the aryl groups of all molecules interacted with the region containing the active-site residues Asp352, Tyr158, Arg442, Glu277, and Tyr72. While the triazole group of the compounds was situated towards the area where the amino acids Pro312, Arg315, Ser311, Asp307, His280 were located, the coumarin ring interacted with the region where the amino acid residues Tyr158, Ser157, Phe178, Glu277, Gln279, Phe303, Ser304, Val308, Arg315, Pro312, His280, Ala329, Asp325, Ser240, Gly309 were located. 2D docking results depicting all other amino acid–ligand interactions are given in Figure S8 for all molecules.

**Figure 5:**
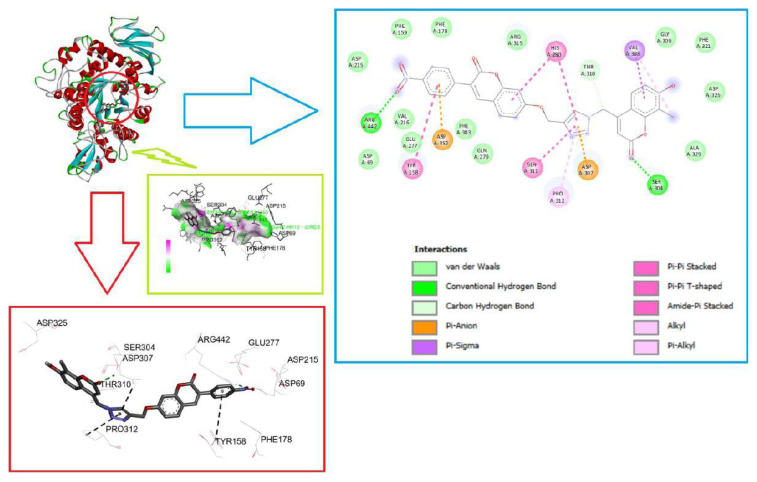
The molecular docking conformation of 4e is shown in the active site of α-glycosidase (PDB No: 3A4A), 3D views of hydrogen bond donor/acceptor surface on the receptor, and 2D view of α-glycosidase interactions.

Before proceeding to the molecular docking calculations of α-glycosidase and the synthesized coumarin thiazole derivatives (4a–4e), the acarbose molecule, a clinically known inhibitor of α-glycosidase, was placed in the binding site, and the binding energy was calculated as – 32.635 kJ/mol. Similar binding calculations were performed for the synthesized compounds, and the binding energies ranged from – 50.626 kJ/mol to – 44.350 kJ/mol. The affinity of all molecules for α-glycosidase is better than the binding energies of the acarbose molecule. As a result of experimental and theoretical studies, the compound thought to have the highest interaction strength was determined to be 4e, and the molecular docking poses of the molecule with the most probable binding conformer are shown in Figure 5. Figure 5 shows the 3D, 2D interaction and hydrogen bond donor/acceptor surface map of the 4e molecule because of the docking study with α-glycosidase. Here, the most effective interaction in the binding mechanism is two conventional hydrogen bonds, and 4e serves as a hydrogen bond acceptor in both. The first of these interactions occurs between the coumarin oxygen atom and HG:SER304, and the other between the oxygen atom of the nitro group and HH:ARG442, with distances of 2.30 and 2.26 Å, respectively. The other most prominent interaction types are π-π interactions between π electrons of the triazole ring and aromatic π electrons of PRO312 protein and carbonyl π electrons of ASP307 protein. The distances of these interactions were measured as 4.53 and 3.70 Å, respectively. The 2D interaction image of all other proteins with which 4e interacts is shown in Figure S7. In addition, it was determined that all calculated molecules (4a–4e) and the reference molecule acarbose also formed hydrogen bonds and hydrophilic interactions with the α-glycosidase enzyme, and the interactions of all molecules are summarized in Table 2. On the other hand, the superimposed structures of acarbose, a standard inhibitor, and the most potent compound, 4e, in the active site are shown in Figure S9. Upon examination, both molecules bind to the same active site of the protein.

In molecular docking calculations, energies obtained solely from molecular mechanics methods are insufficient. Furthermore, factors affecting IC_50_, such as solvent effects, cell permeability, and metabolic stability, are not included in the theoretical calculations. When all these factors are considered, some differences in the calculations emerge. However, when we evaluate the IC_50_ and molecular docking calculations separately, we observe a partial correlation. The molecule with the highest binding energy is molecule 4e, at – 50.626 kJ/mol, while the molecule with the lowest IC_50_ is also 4e. It can be said that molecules 4b and 4d are excluded from this correlation. However, for the reasons stated above, this situation is considered acceptable.

### 3.5. In silico ADME and drug-likeness prediction

Nowadays, ADME studies are used in drug production with molecular docking calculations to select the most promising compounds with the highest docking binding energy and to minimize the risk of late-stage drug attrition. With these studies, the balance between pharmacodynamic and pharmacokinetic properties can be determined as preliminary information [49]. Here, various parameters, including molecular properties, drug solubility (S), cell permeability, Human Intestinal Absorption HIA, polar surface area (PSA), and drug similarity score, are investigated using virtual screening methods on small molecules. According to Lipinski’s rule of five, the molecular weight and LogP of an existing oral drug selected should not exceed 500 and five, respectively. They should have fewer than ten hydrogen bond acceptors and fewer than five hydrogen bond donors. In addition, the Topological PSA value is expected to be less than 140. In this study, online servers such as SwissADME were used in all calculations.

The physicochemical properties and Lipinski parameters of all synthesized molecules were calculated and are listed in Table 3. When the results in Table 3 are examined, the molecular weights of all compounds are slightly above 500 g/mol. LogP values of all molecules are 2.38–3.36 (< 5), acceptor hydrogen bond (AHB) 7–9 (< 10), donor hydrogen bond (DHB) 3–5 (< 5), and are compatible. Topological PSA values were calculated between 113.27–122.5 < 140 in 4a–4d molecules and 166.09 A2 in 4e (Figure S10). The ABS values of the molecules ranged from 51.70% to 69.92%.

The properties of all compounds, including predicted water solubility, predicted pharmacokinetic (ADME, i.e., absorption, distribution, metabolism, excretion) parameters, predicted drug similarity, medicinal chemistry, and lead similarity pharmacokinetic parameters, were also calculated and are given in Table 4.

Solubility is an important criterion in drug absorption. When all compounds were evaluated on this scale, it was determined that they had poor solubility, with 4c and 4e having slightly more solubility. It was determined that the molecules examined in the study, except for 4e, exhibited high gastrointestinal (GI) absorption and crossed the blood–brain barrier (BBB). Although compound 4e showed strong binding affinity and a low IC_50_, its GI absorption was underestimated due to the nitro group’s polarity and metabolic risks. Another pharmacokinetic property, skin permeation parameters, was calculated according to the criteria defined by Potts and Guy. These criteria state that skin permeation decreases as the LogK value increases, and the values of the synthesized compounds are listed in Table 4. Finally, 4b–4e, which were synthesized, did not inhibit 4 of the five important enzymes of the cytochrome P450 (CYP) system, which has an important role in the degradation of drug candidates excreted from the body, and it was determined that it inhibited the CYP2C9 enzyme. 4a did not inhibit any of the five enzymes. This result may reduce the rate of drug metabolism via these compounds, leading to accumulation in the body, increased plasma levels, increased risk of toxicity, and increased potential for interactions with other drugs.

When key ADME parameters, such as Lipinski’s rule of five, water solubility, and potential hepatotoxicity, are evaluated together, compound 4a appears to perform best among the compounds, offering a balanced profile. It can be said to have the advantage of drug similarity due to its high GI absorption, acceptable lipophilicity, and moderate solubility. Compound 4e, on the other hand, is the strongest candidate in terms of biological activity, but it suffers from low absorption, high TPSA, CYP2C9 inhibition, and multiple violations of the TPSA regulation. Therefore, structural optimization (e.g., reducing TPSA and molecular weight) may be recommended to increase the bioavailability of compound 4e to enhance its pharmacological effect.

As a result of the in silico studies, it was determined that the molecules complied with most of the Lipinski rules, exhibited poor water solubility, and possessed appropriate pharmacokinetic and drug-like properties. Although all compounds slightly exceeded the 500 g/mol threshold for molecular weight, they complied with other key Lipinski parameters, suggesting acceptable oral bioavailability potential.

## 4. Conclusion

In this work, the α-glycosidase inhibitory effects of the compounds were examined using a spectrophotometric assay. 4e exhibited the best inhibitory effects with an IC_50_ value of 38.98 ± 0.77 μM on α-glycosidase. The kinetic analysis indicated that 4e was a mixed inhibitor of α-glycosidase, with a Ki value of 19.95 ± 0.15 μM. These experimental results are also supported by molecular docking and ADME calculations.

## Supplementary Information



## Figures and Tables

**Table 1:** Inhibitory effects of the compounds on α-glycosidase.

	IC_50_ (μM)	R^2^	Equation	Type	*K_i_* (μM)
**4a**	95.04 ± 3.55	0.9969	y = 0.4865x − 3.7619	-	-
**4b**	122.57 ± 4.20	0.9757	y = 0.3705x + 4.5873	-	-
**4c**	108.97 ± 2.40	0.9891	y = 0.5348x − 8.2768	-	-
**4d**	93.55 ± 1.70	0.9599	y = 0.5178x + 1.5577	-	-
**4e**	38.98 ± 0.77***	0.98	y = 0.6523x + 24.573	Mixed	19.95 ± 0.15
**Acarbose**	64.49 ± 2.53	0.9806	y = 0.6127x + 10.482	-	-

*** p < 0.0001 vs. acarbose

**Table 2:** Protein–ligand interactions and docking scores of compounds^a^.

Compounds	Docking energy (kJ/mol)	Protein–ligand interactions			

		Ligand	Receptor atoms	Interaction type	Distance (Å)
**4a**	−48.116	OH(coumarin)	O:SER157	HBD	1.91
		N (triazole)	NH:ARG315	HBA	2.81
		O(coumarin)	HE:GLN279	HBA	2.39
		Ring (coumarin)	Ring:PHE303	p-p	5.01
**4b**	−47.279	O(coumarin)	NH:ARG442	HBA	2.32
		O(coumarin)	HG:SER304	HBA	1.88
		Ring (aryl)	C=O:ASP352	p-p	4.24
		Ring (triazole)	C=O:ASP307	p-p	3.58
**4c**	−44.350	O(coumarin)	HD:HIS280	HBA	1.92
		Ring (triazole)	C=O:ASP307	p-p	3.88
		Ring (coumarin)	C=O:ASP307	p-p	3.97
**4d**	−46.442	O(coumarin)	HE:GLN279	HBA	2.72
		O(coumarin)	HE:GLN279	HBA	2.25
		OH(coumarin)	O:TYR158	HBD	1.87
		Ring (coumarin)	Ring:GLN279	p-p	4.96
**4e**	−50.626	O(coumarin)	HG:SER304	HBA	2.30
		O(NO2)	HH:ARG442	HBA	2.16
		Ring (triazole)	Ring:PRO312	p-p	4.53
		Ring (triazole)	C=O:ASP307	p-p	3.70
Acarbose	−32.635	OH	O:PRO312	HBD	1.92
		OH	O:LEU313	HBD	2.92
		O	HZ3,:LYS156	HBA	2.13
		O	HZ2,:LYS156	HBA	2.40
		O	HZ3,:LYS156	HBA	2.80
		O	HN:SER240	HBA	2.55
		OH	O:ASP242	HBD	2.11
		NH	O:ASP307	HBD	2.61

a HBA = hydrogen bond acceptor, HBD = hydrogen bond donor.

**Table 3:** Physicochemical and lipophilicity of the compounds.

Compounds	Lipophilicity consensus log P	Physicochemical properties								
		MA^a^ g/mol	Heavy atoms	Aromatic heavy atoms	Rot. bond	H-bond acc.	H-bond don.	MR^b^	TPYA^c^ (A^2^)	% ABS^d^
**4a**	3.20	514.55	38	16	5	7	3	153.69	113.27	69.92
**4b**	3.36	528.58	39	16	5	7	3	158.50	113.27	69.92
**4c**	2.74	544.58	40	16	6	8	3	159.58	122.50	66.74
**4d**	3.20	532.54	39	16	5	8	3	153.74	113.27	69.92
**4e**	2.38	556.52	41	16	5	9	5	158.94	166.09	51.70

a **MA**, molecular weight;

b **MR**, molar refractivity;

c **TPYA**, topological polar surface area;

d **ABS%:** absorption percentage (ABS% = 109 − [0.345 × TPYA]).

**Table 4:** Predicted water solubility, predicted pharmacokinetics (ADME) parameters, predicted drug-likeness, medicinal chemistry, and lead-likeness pharmacokinetics parameters of compounds.

Compounds Phy.chem.prprt.	4a	4b	4c	4d	4e
***Water solubility***					
ESOL log S	−6.48	−6.28	−5.96	−6.27	−5.98
ESOL solubility (mg/ml)	1.72e-4	2.78e-4	6.00e-4	2.89e-4	5.78e-4
ESOL class	Poorly soluble	Poorly soluble	Moderately soluble	Poorly soluble	Moderately soluble
***Pharmacokinetics***					
GI absorption	High	High	High	High	Low
BBB permeant	No	No	No	No	No
Pgp substrate	Yes	Yes	Yes	Yes	No
CYP1A2 inhibitor	No	No	No	No	No
CYP2C19 inhibitor	No	No	No	No	No
CYP2C9 inhibitor	No	Yes	Yes	Yes	Yes
CYP2D6 inhibitor	No	No	No	No	No
CYP3A4 inhibitor	No	No	No	No	No
Log Kp (cm/s)	−5.53	−5.93	−6.42	−6.00	−6.61
***Druglikeness***					
Lipinski #violations	1	1	1	1	2
Ghose #violations	2	2	2	2	2
Veber #violations	0	0	0	0	1
Bioavailability score	0.55	0.55	0.55	0.55	0.17

## Footnotes

1 RCSB Protein Data Bank (2025). RCSB PDB: Resources for Studying Biological Macromolecules [online]. Website: https://www.rcsb.org/ [accessed 20 June 2025].

2 SwissADME (2025). SwissADME: A Free Web Tool to Evaluate Pharmacokinetics, Drug-Likeness and Medicinal Chemistry Friendliness of Small Molecules [online]. Website: http://www.swissadme.ch/index.php [accessed 20 June 2025].
