# Supplementary material for: α-Glycosidase activity of novel coumarin–triazole–coumarin dyads
Source: Turk J Chem. 2025 Oct 27;49(6):780–92. doi: 10.55730/1300-0527.3770 (PMC12779026; doi:10.55730/1300-0527.3770)
Supplement: Supplementary file 1 [file 49-6-00304-supp.docx]

**Supporting Information**

**α-Glycosidase activity of novel coumarin-triazole-coumarin dyads**

Ersin ŞİRİN^1^, Esra SEVİMLİ^1^, Gökçe SEYHAN^2^, Burak BARUT^2^, Yunus KAYA^1^, Baybars KÖKSOY^1*^

^1^Department of Chemistry, Faculty of Engineering and Natural Sciences, Bursa Technical University, Bursa, Turkey.

^2^Department of Biochemistry, Faculty of Pharmacy, Karadeniz Technical University, Trabzon, Turkey

*Correspondence: Assoc. Prof. Dr. Baybars KÖKSOY – [baybars.koksoy@btu.edu.tr](mailto:baybars.koksoy@btu.edu.tr)

ORCIDs:

Ersin ŞİRİN: https://orcid.org/0000-0003-2034-0734

Esra SEVİMLİ: https://orcid.org/0000-0002-7935-3954

Gökçe SEYHAN: https://orcid.org/0000-0002-8553-9093

Burak BARUT: https://orcid.org/0000-0002-7441-8771

Yunus KAYA: https://orcid.org/0000-0001-7228-2340

Baybars KÖKSOY: https://orcid.org/0000-0001-7939-5380

**1.Synthesis and characterization of starting materials**

**1.1 Synthesis of alkyne-coumarins (2a-2e)**

Alkyne coumarin derivatives (**1a-1e**) were prepared using the reported methods [25–28]. 3,5 mmol **1a-1e** (833,84 mg **1a**, 882,95 mg **1b**, 938,95 mg **1c**, 896,80 mg **1d**, and 991,34 mg **1e**) and propargyl bromide (5,25 mmol, 624.54 mg) were dissolved in 40 mL dry DMF. Afterward, anhydrous K_2_CO_3_ (5,25 mmol, 725,55 mg) was added to the reaction medium and stirred for 48 h at 65˚C. After completion, the reaction was cooled down and poured into the ice-water. The precipitate was filtered and washed with water. The crude product was purified by column chromatography using a solvent chloroform.

**1.1.1 3-Phenyl-7-(prop-2-yn-1-yloxy)-2*H*-chromen-2-one (2a)**

Light yellow solid, Yield: 81%, m.p. 147-150˚C. FT-IR (ATR, cm^-1^): 3281 (ʋC≡C-H), 3052 (ʋArC-H), 2925 (ʋAlC-H), 2119 (ʋC≡C), 1720 (ʋC=O), 1604 (ʋC=C). ^1^H NMR (500 MHz, CDCl_3_) δ (ppm): 7.80 – 7.73 (m, 1H), 7.67 (d, *J* = 10.8 Hz, 2H), 7.43 (q, *J* = 22.5, 15.6 Hz, 4H), 6.96 (d, *J* = 26.6 Hz, 2H), 4.78 (t, *J* = 5.5 Hz, 2H), 2.58 (s, 1H), CHN Analysis. Calcd for C_18_H_12_O_3_ (276,08 g/mol): C, 78,25; H, 4,38; Found C, 78,23; H, 4,36.

**1.1.2 7-(Prop-2-yn-1-yloxy)-3-(*p*-tolyl)-2*H*-chromen-2-one (2b)**

Light yellow solid, Yield: 85%, m.p. 160-164˚C. FT-IR (ATR, cm^-1^): 3253 (ʋC≡C-H), 3007 (ʋArC-H), 2943 (ʋAlC-H), 2127 (ʋC≡C), 1729 (ʋC=O), 1611 (ʋC=C). ^1^H NMR (500 MHz, CDCl_3_) δ (ppm): 7.76 (d, *J* = 1.8 Hz, 1H), 7.61 (dd, *J* = 8.1, 2.1 Hz, 2H), 7.47 (dd, *J* = 8.5, 2.0 Hz, 1H), 7.29 – 7.25 (m, 2H), 7.00 (d, *J* = 2.6 Hz, 1H), 6.95 (dt, *J* = 8.7, 2.2 Hz, 1H), 4.80 (d, *J* = 2.4 Hz, 2H), 2.61 (q, *J* = 2.3 Hz, 1H), 2.42 (s, 3H), CHN Analysis. Calcd for C_19_H_14_O_3_ (290,09 g/mol): C, 78,61; H, 4,86; Found C, 78,60; H, 4,85.

**1.1.3 3-(4-Methoxyphenyl)-7-(prop-2-yn-1-yloxy)-2*H*-chromen-2-one (2c)**

Cream solid, Yield: 73%, m.p. 180˚C (decomposed). FT-IR (ATR, cm^-1^): 3251 (ʋC≡C-H), 3010 (ʋArC-H), 2946 (ʋAlC-H), 2126 (ʋC≡C), 1728 (ʋC=O), 1611 (ʋC=C). ^1^H NMR (400 MHz, CDCl_3_) δ (ppm): 7.71 (s, 1H), 7.64 (d, *J* = 8.2 Hz, 2H), 7.44 (d, *J* = 8.4 Hz, 1H), 7.08 – 6.86 (m, 4H), 4.76 (s, 2H), 3.84 (s, 3H), 2.58 (s, 1H), CHN Analysis. Calcd for C_19_H_14_O_4_ (306,32 g/mol): C, 74,50; H, 4,61; Found C, 74,48; H, 4,58.

**1..1.4 3-(4-Fluorophenyl)-7-(prop-2-yn-1-yloxy)-2*H*-chromen-2-one (2d)**

Cream solid, Yield: 80%, m.p. 177-180˚C. FT-IR (ATR, cm^-1^): 3291 (ʋC≡C-H), 3048 (ʋArC-H), 2920 (ʋAlC-H), 2121 (ʋC≡C), 1700 (ʋC=O), 1616 (ʋC=C). ^1^H NMR (500 MHz, CDCl_3_) δ (ppm): 7.77 (d, *J* = 2.1 Hz, 1H), 7.70 (ddd, *J* = 8.2, 5.6, 2.1 Hz, 2H), 7.49 (dd, *J* = 8.7, 2.0 Hz, 1H), 7.15 (td, *J* = 8.7, 2.2 Hz, 2H), 7.01 (d, *J* = 2.5 Hz, 1H), 6.97 (dd, *J* = 8.7, 2.5 Hz, 1H), 4.80 (t, *J* = 2.4 Hz, 2H), 2.61 (q, *J* = 2.5 Hz, 1H), CHN Analysis. Calcd for C_18_H_11_FO_3_ (294,28 g/mol): C, 73,47; H, 3,77; Found C, 73,45; H, 3,75.

**1.1.5 3-(4-Nitrophenyl)-7-(prop-2-yn-1-yloxy)-2*H*-chromen-2-one (2e)**

Yellow solid, Yield: 72,4%, m.p. 245˚C (decomposed). FT-IR (ATR, cm^-1^): 3284 (ʋC≡C-H), 3068 (ʋArC-H), 2924 (ʋAlC-H), 2129 (ʋC≡C), 1714 (ʋC=O), 1611 (ʋC=C). ^1^H NMR (500 MHz, DMSO-d6) δ (ppm): 8.41 (s, 1H), 8.28 (d, *J* = 8.8 Hz, 2H), 8.01 – 7.97 (m, 2H), 7.74 (d, *J* = 8.7 Hz, 1H), 7.10 (d, *J* = 2.7 Hz, 1H), 7.03 (dd, *J* = 8.7, 2.7 Hz, 1H), 4.95 (d, *J* = 2.4 Hz, 2H), 3.65 (t, *J* = 2.7 Hz, 1H), CHN Analysis. Calcd for C_18_H_11_NO_5_ (321,29 g/mol): C, 67,29; H, 3,45; N, 4,36; Found C, 67,26; H, 3,43; N, 4,35

**1.2 Synthesis of azide-coumarin (3b)**

Coumarin derivative **3a** (1130 g, 5,9 mmol) was dissolved in acetone. Then sodium azide (423 mg, 6,5 mmol) was added to the reaction mixture and heated to 60˚C. After 48 h, the reaction mixture was poured into ice-water, filtered and washed with water. The crude product was recrystallized from ethanol.

Brown solid, Yield: 80%, m.p. 140˚C (decomposed). FT-IR (ATR, cm^-1^): 3063 (ʋArC-H), 2938 (ʋAlC-H), 2108 (N_3_), 1703 (ʋC=O), 1602 (ʋC=C). ^1^H NMR (400 MHz, DMSO-d6) δ (ppm): 10.52 (s, 1H), 7.37 (d, *J* = 8.7 Hz, 1H), 6.84 (d, *J* = 9.0 Hz, 1H), 6.24 (s, 1H), 4.74 (s, 2H), 2.12 (s, 3H), CHN Analysis. Calcd for C_11_H_9_N_3_O_3_ (231,21 g/mol): C, 57,14; H, 3,92; N, 18,17; Found C, 57,13; H, 3,91; N, 18,15.

**DFT Studies**

Geometry optimizations and vibrational frequency calculations of the experimentally synthesized compounds 4a–e were performed using Density Functional Theory (DFT) at the B3LYP level with the 6-31G(d,p) basis set. The absence of imaginary frequencies confirms that the optimized structures correspond to true minimum on the potential energy surface. The optimized structures of the molecules are depicted in Figure S8.

The frontier molecular orbital energies (E_HOMO_ and E_LUMO_), band gap (ΔE), dipole moment, and polarizability values of the optimized compounds are summarized in Table S1. Among the series, compound 4e exhibits the lowest HOMO and LUMO energies, as well as the highest dipole moment and polarizability, indicating enhanced electronic reactivity and polar character.

Notably, compound 4e also demonstrates the most favorable binding energy and the lowest experimentally determined IC50 value, suggesting superior inhibitory activity. A positive correlation was observed between EHOMO and ΔE values and the IC50 data, implying that increased HOMO energy and band gap are associated with decreased IC50 values and improved binding affinity. On the other hand, compound 4e, which exhibits the highest affinity, has the highest dipole moment and polarizability. In other compounds, a partial relationship can be established between dipole moment or polarizability and binding energy or IC50 values. We can say that as the dipole moment or polarizability increases, the IC50 value decreases. These calculated data demonstrate that electronic properties directly affect biological activity and that these parameters should be considered in molecular design.

**Figure S1.** Synthetic route of starting materials.


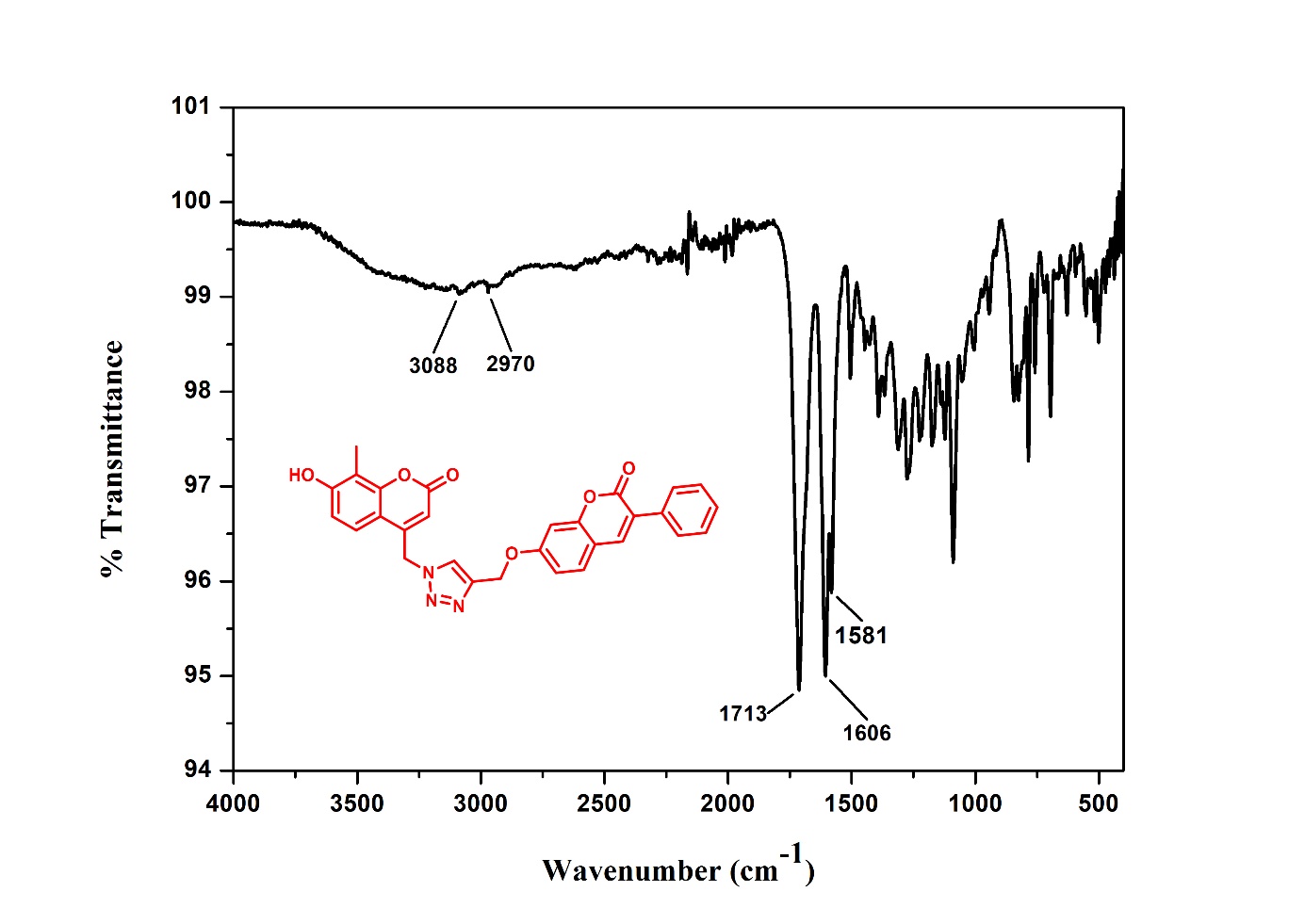


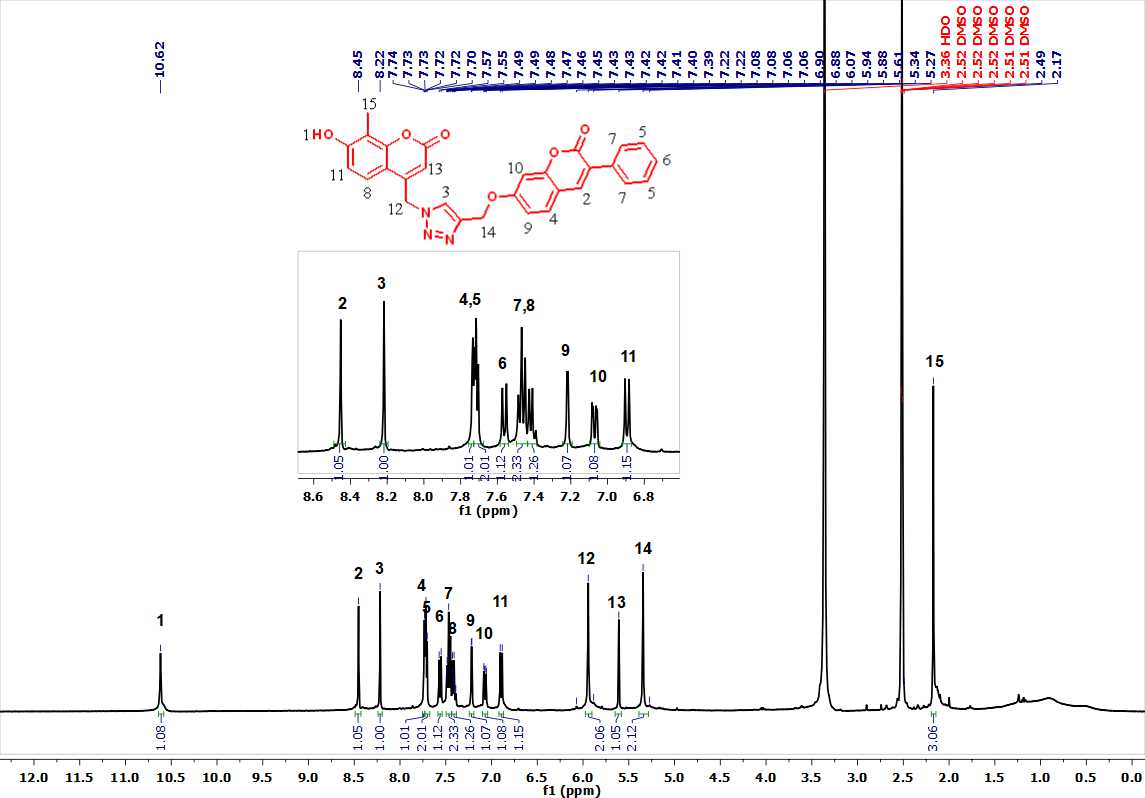


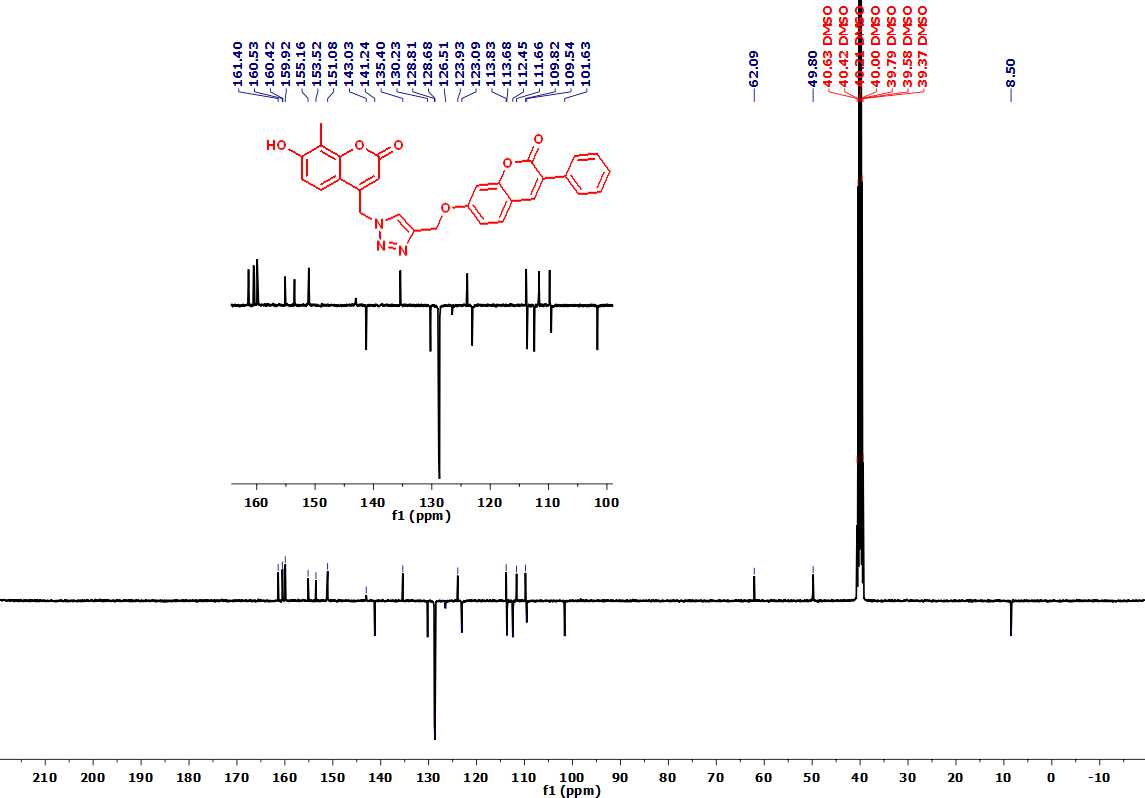


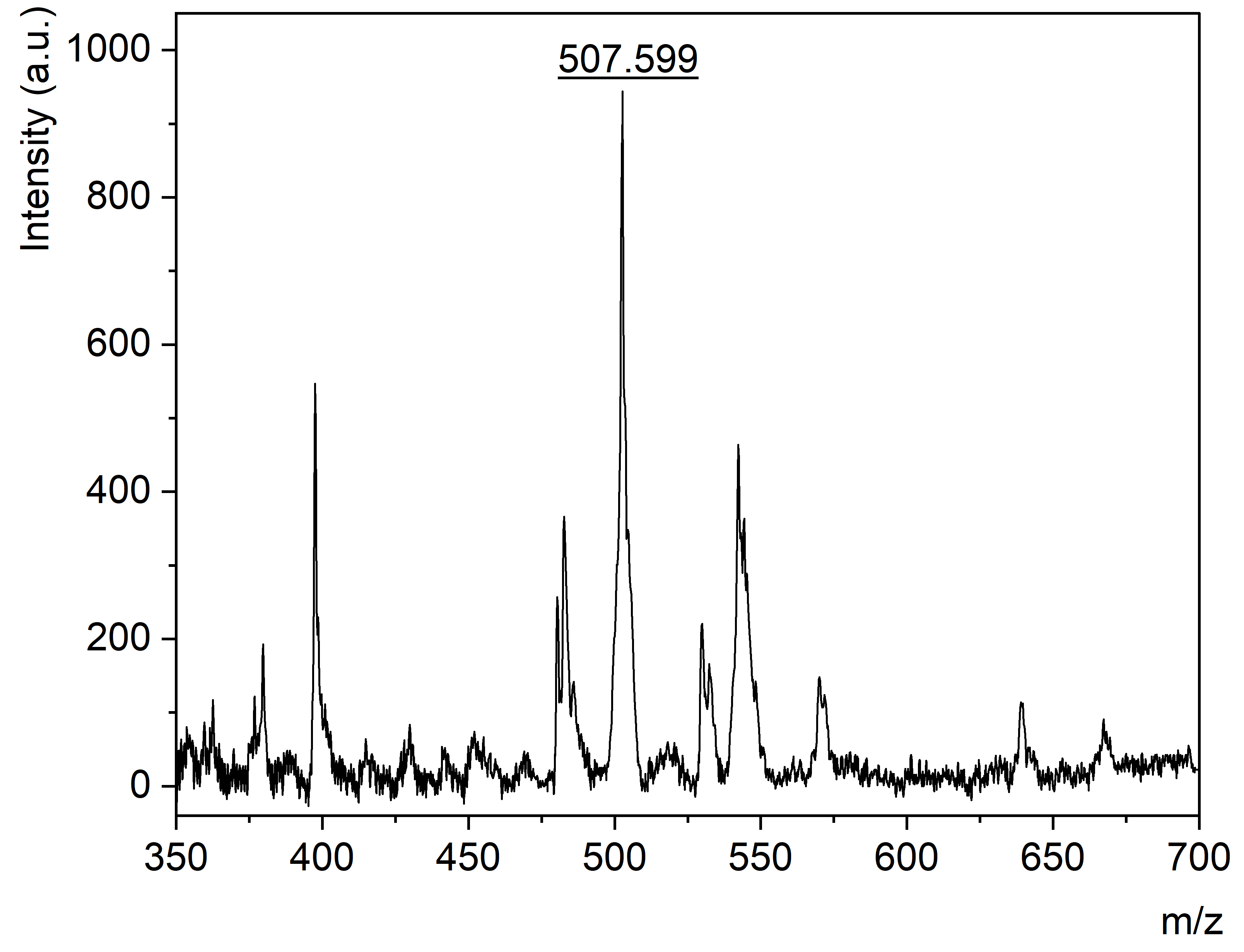


**Figure S2:** FT-IR, ^1^H and ^13^C NMR spectrum and MALDİ-TOFF Mass spectra of compound 4a.


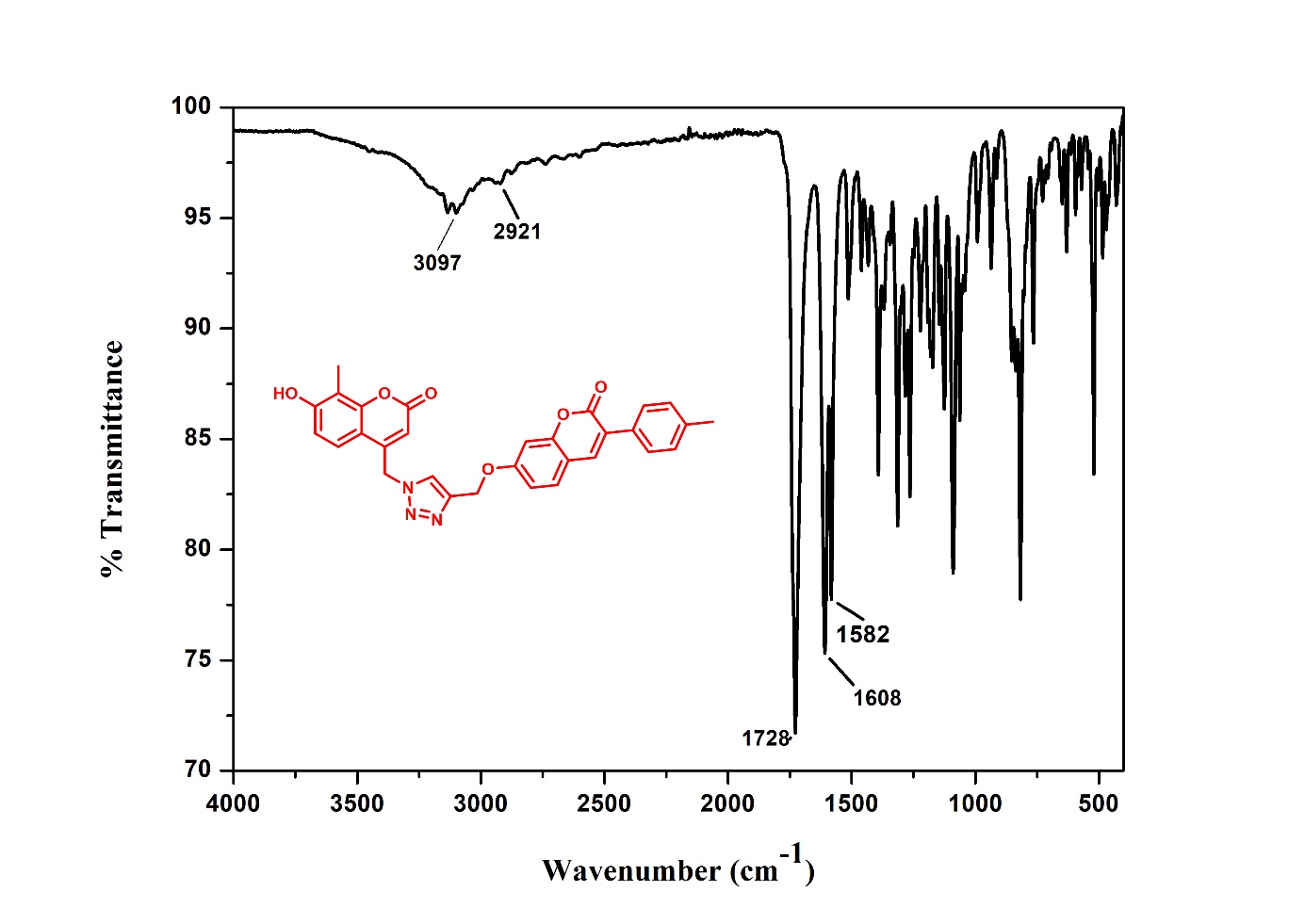


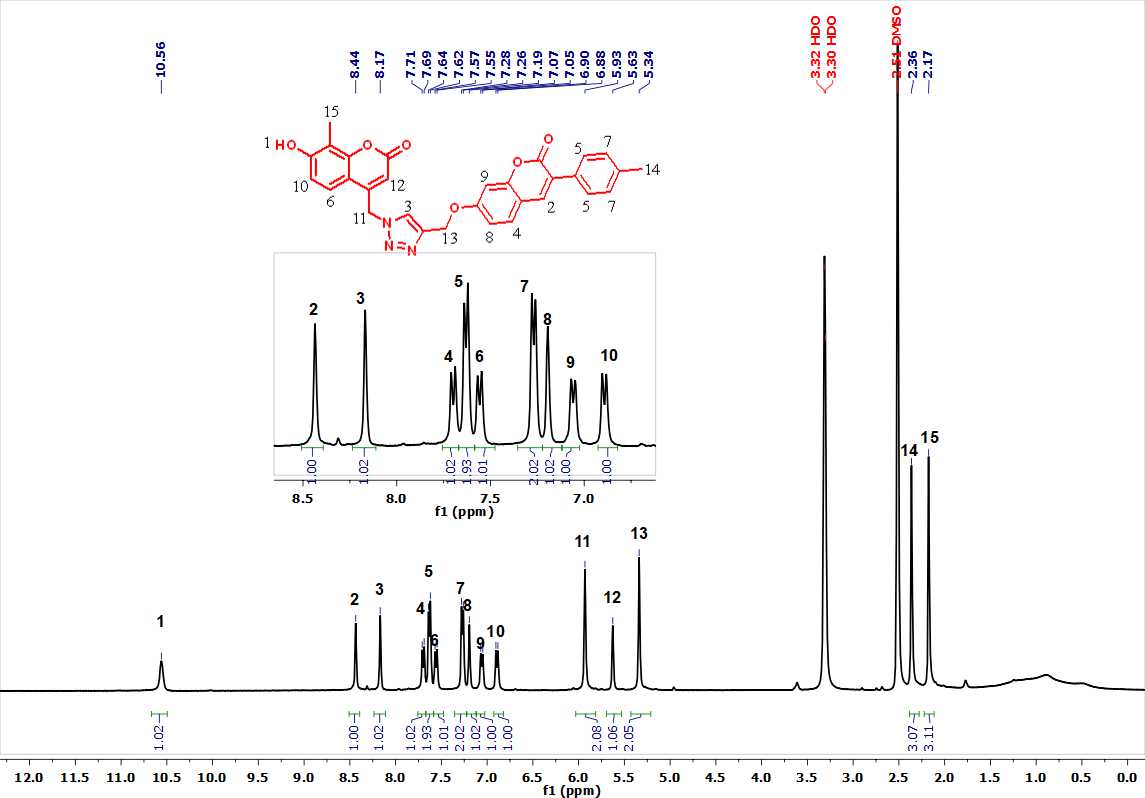


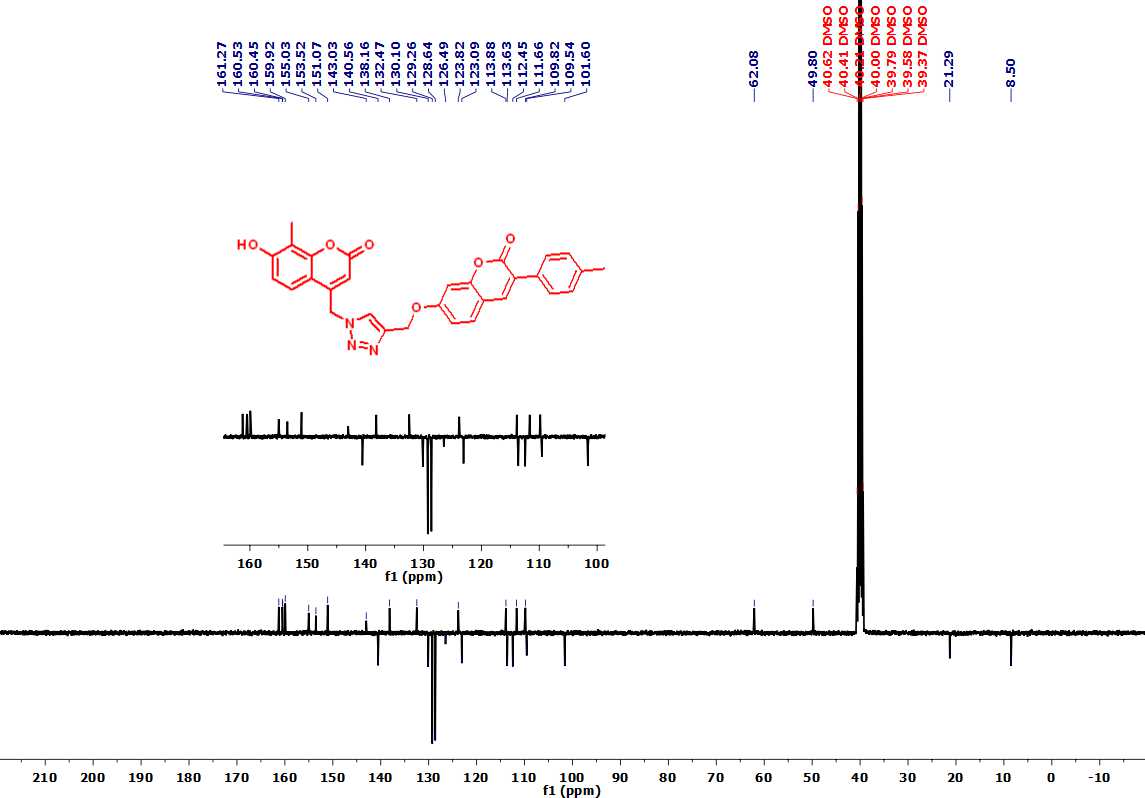


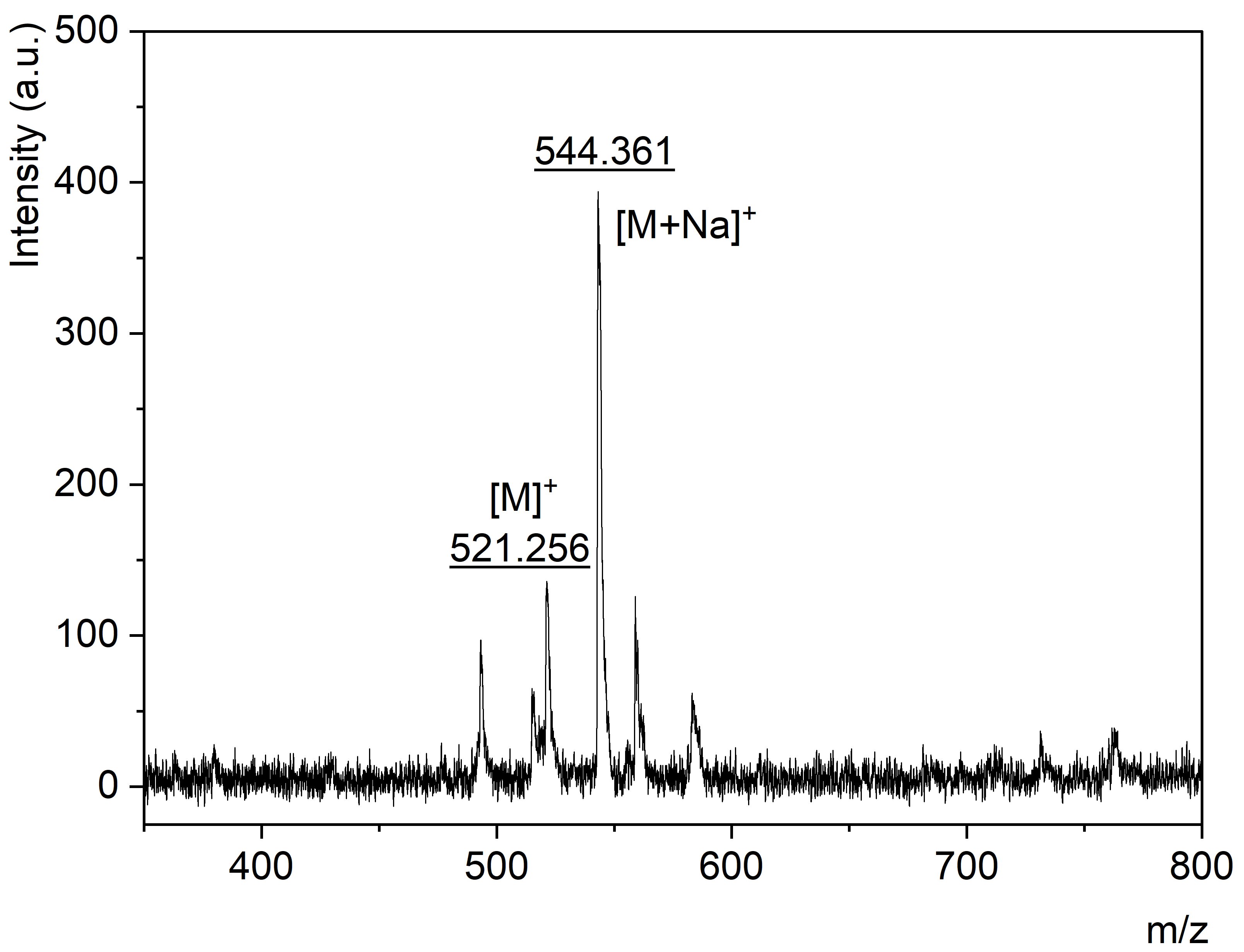


**Figure S3:** FT-IR, ^1^H and ^13^C NMR spectrum and MALDİ-TOFF Mass spectra of compound 4b.


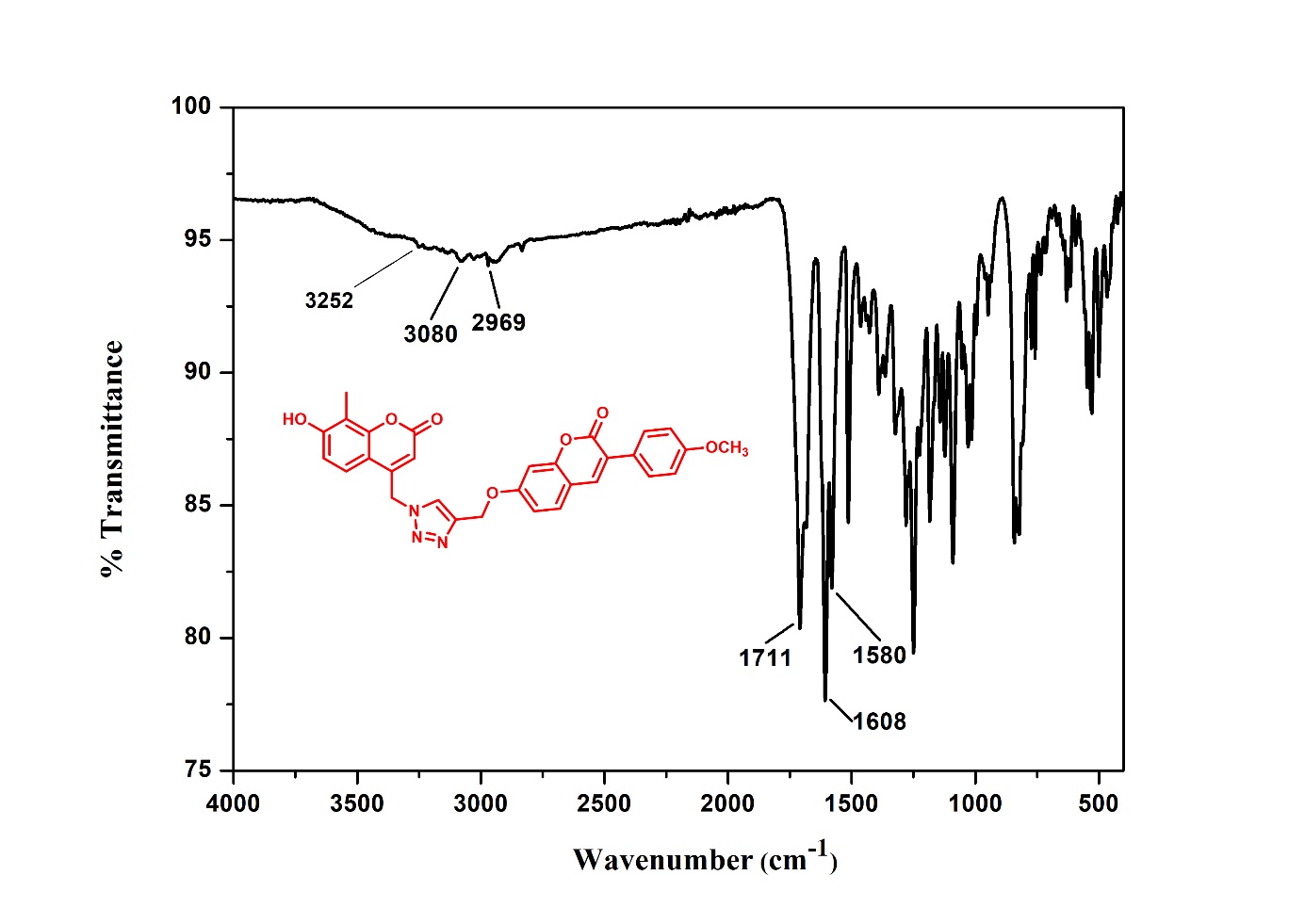


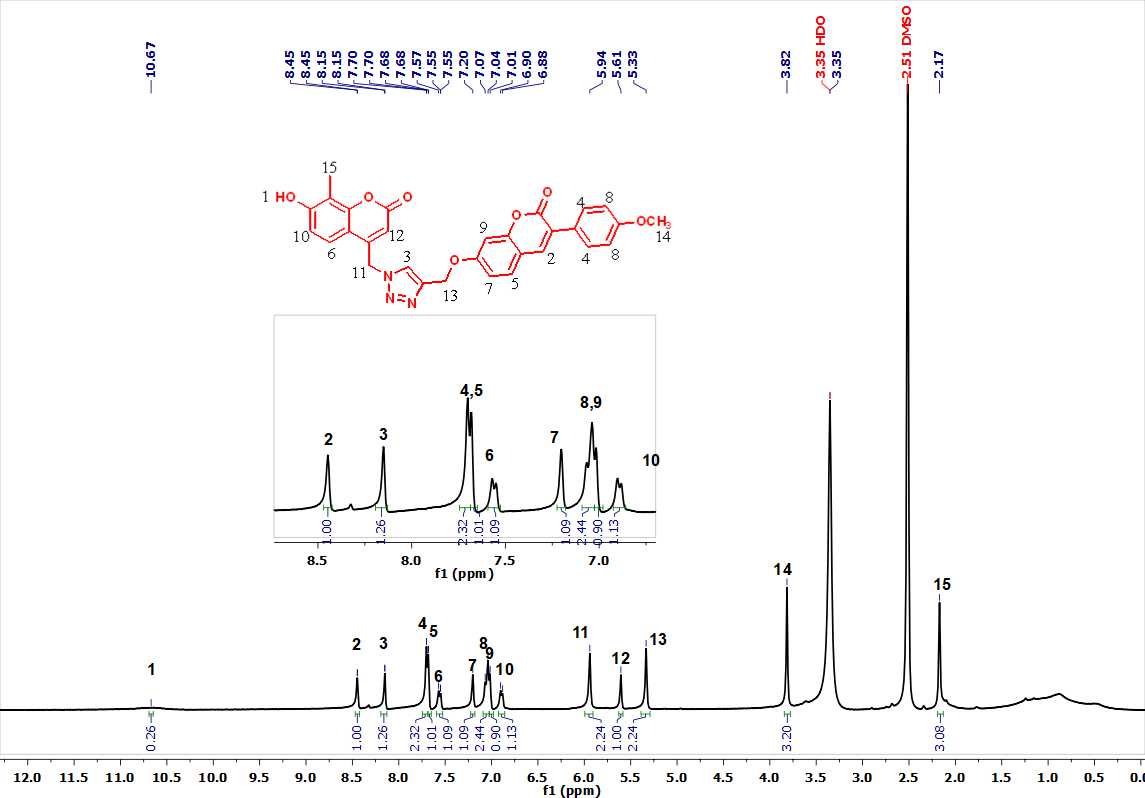


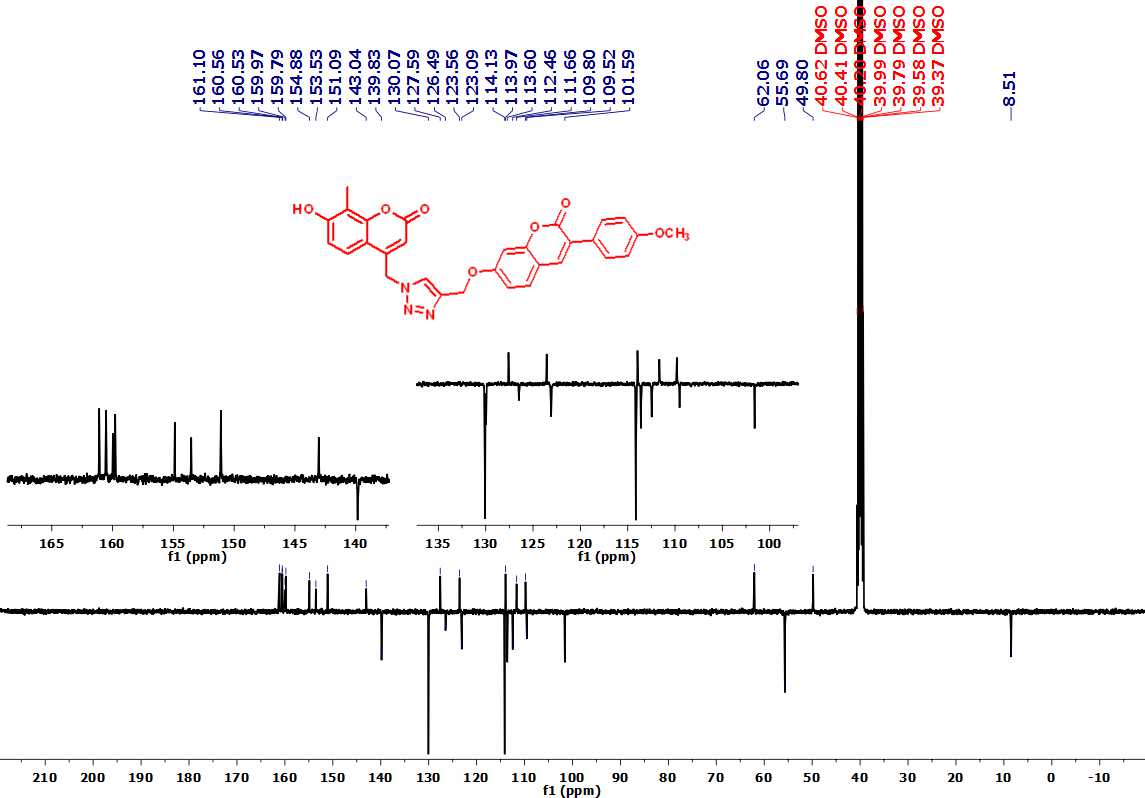


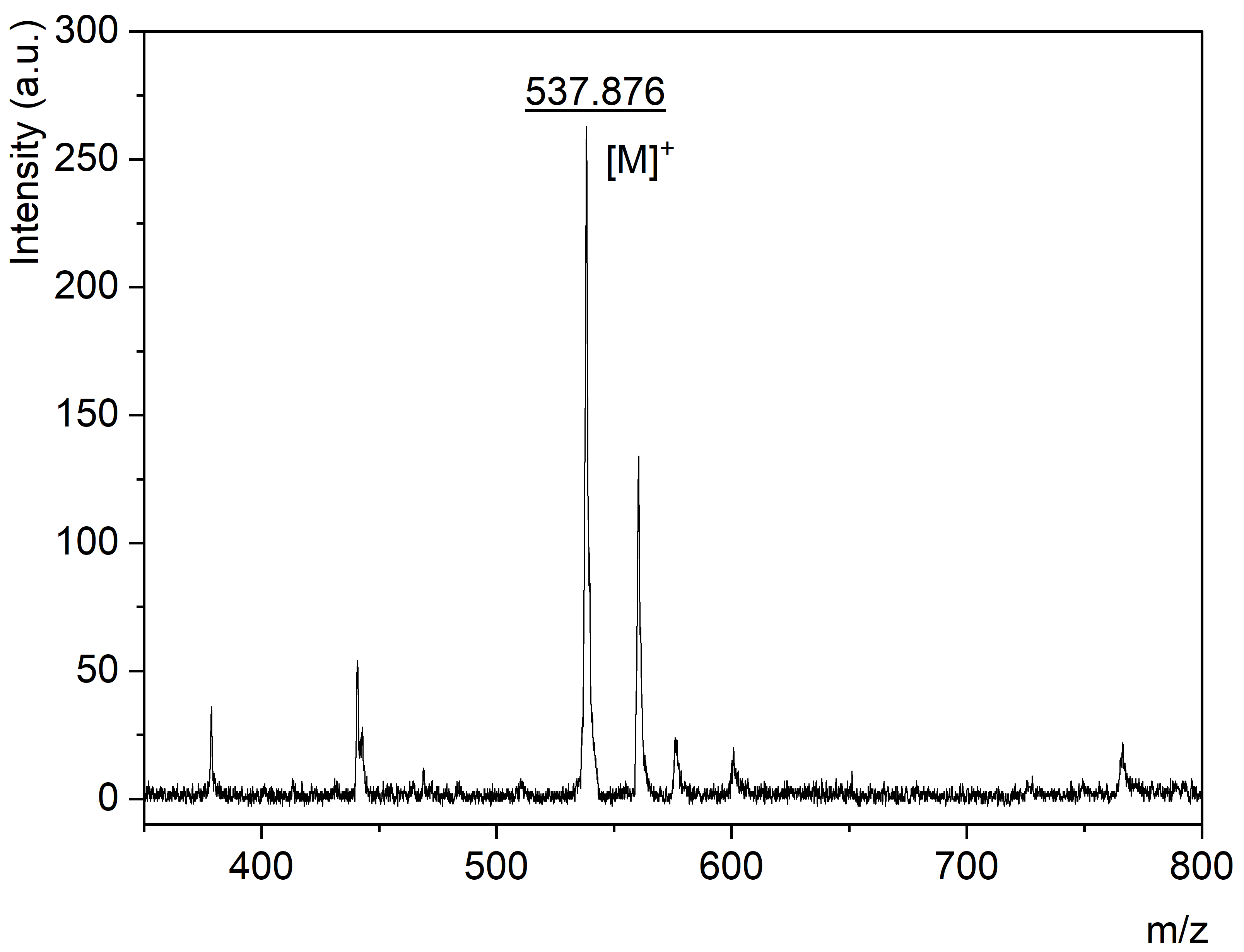


**Figure S4:** FT-IR, ^1^H and ^13^C NMR spectrum and MALDİ-TOFF Mass spectra of compound 4c.


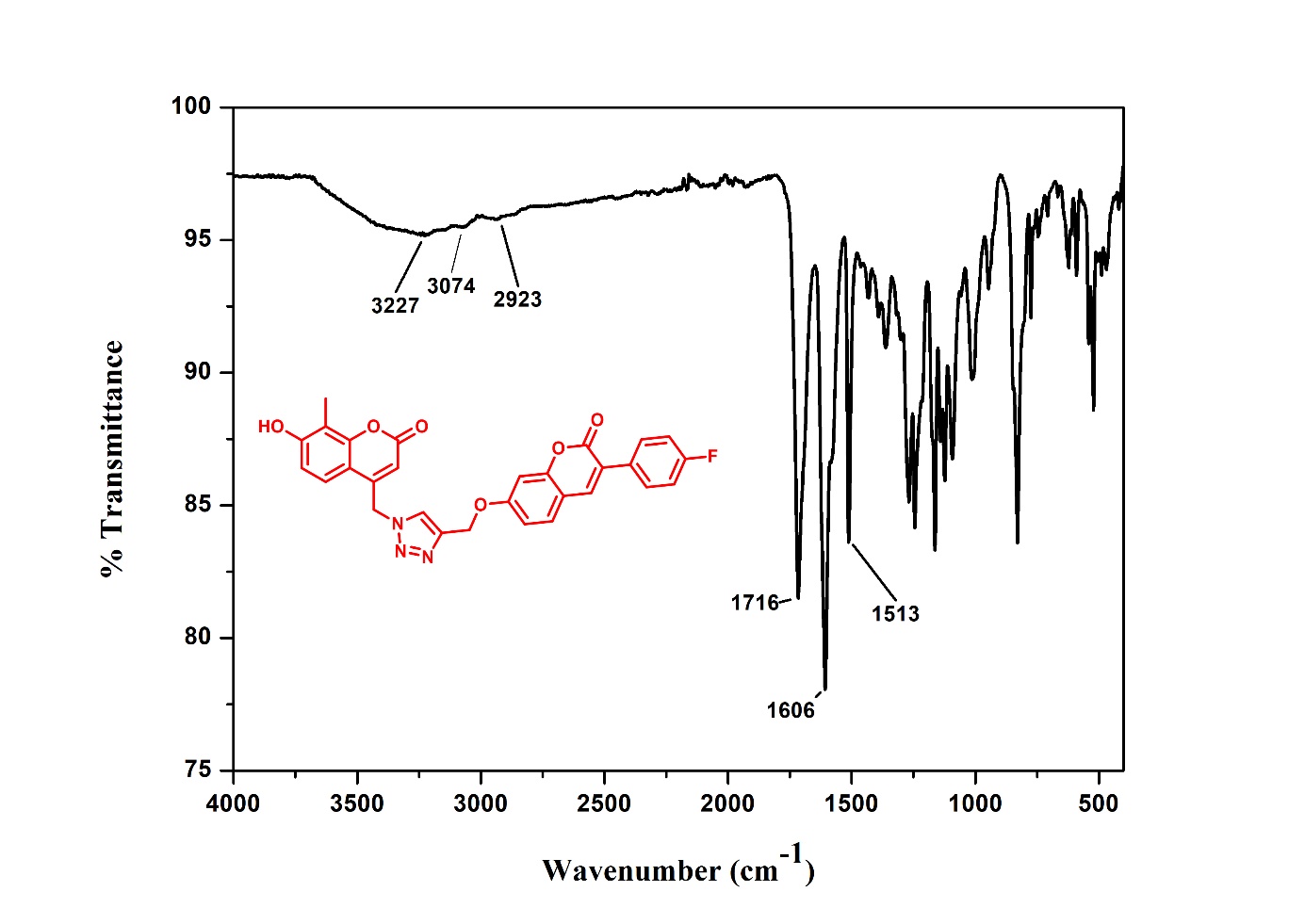


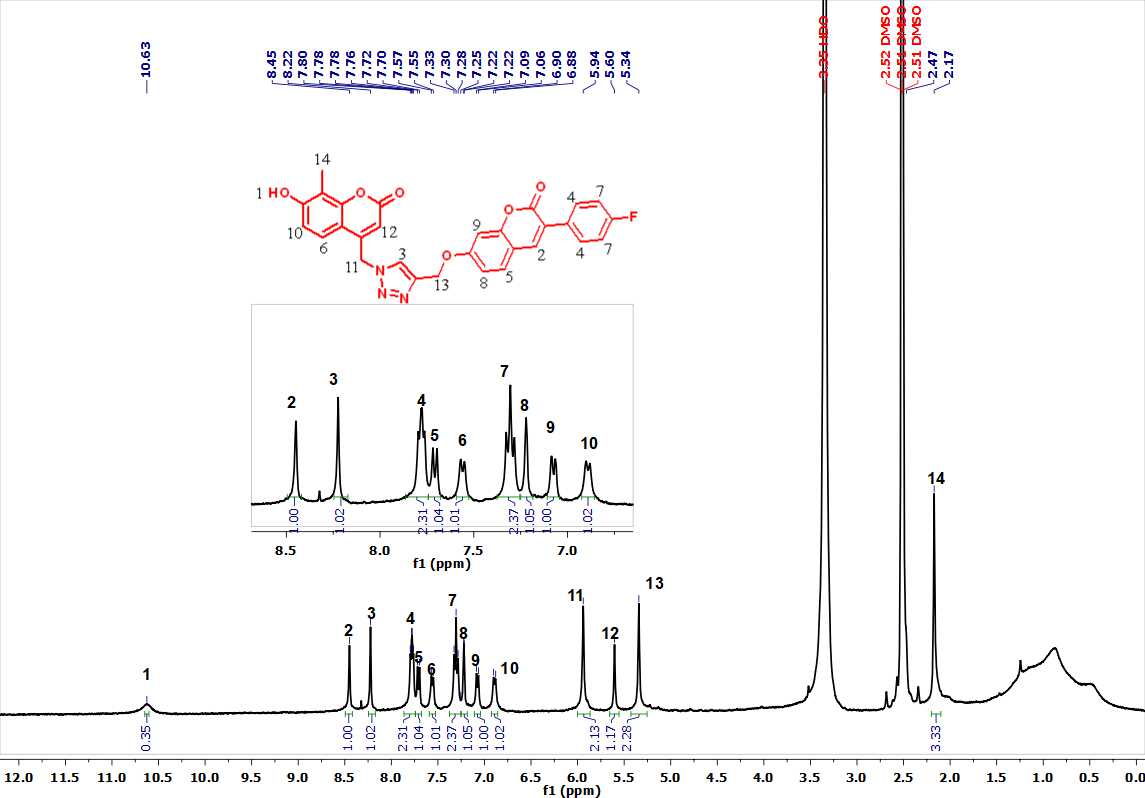


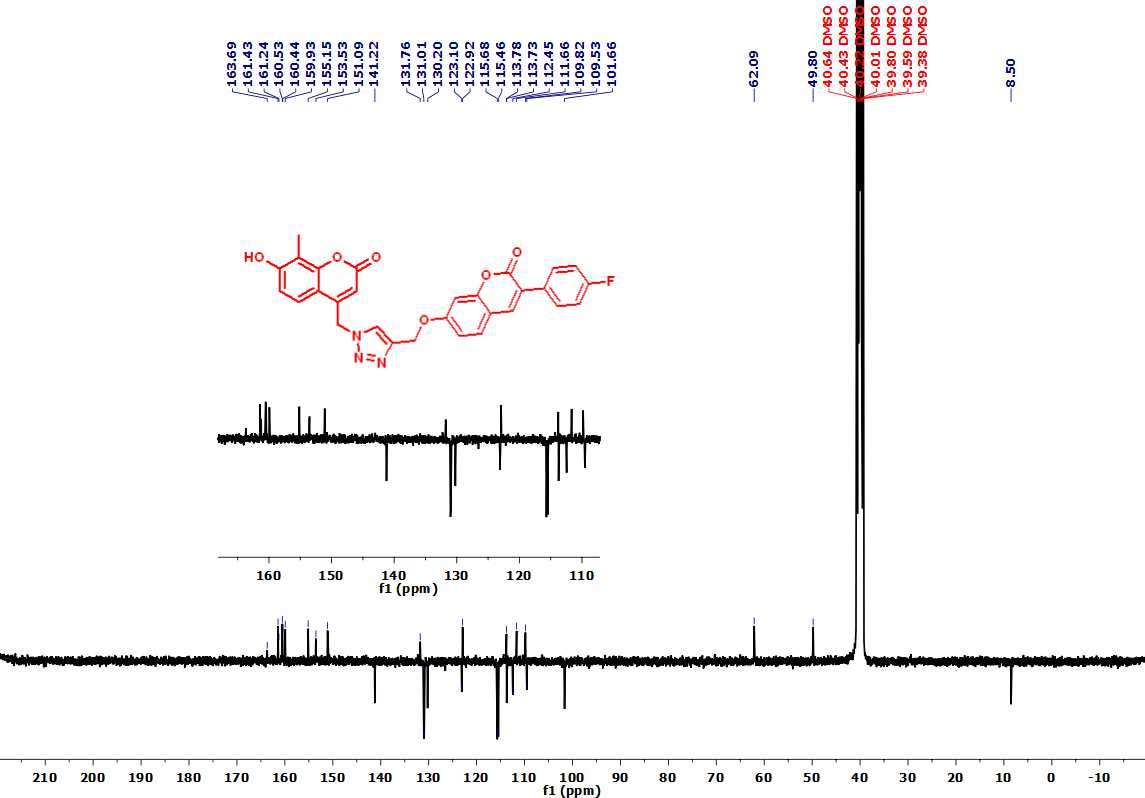


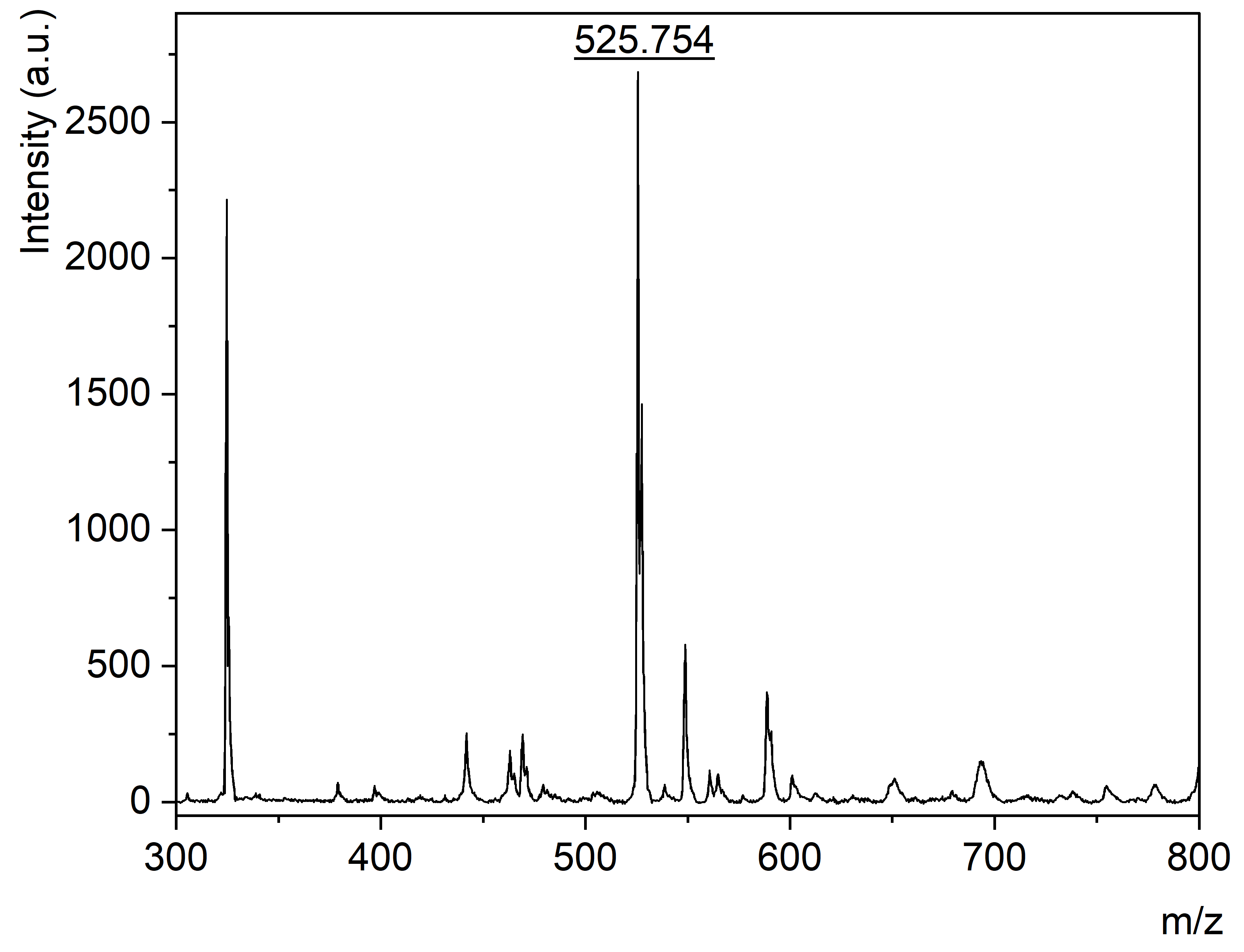


**Figure S5:** FT-IR, ^1^H and ^13^C NMR spectrum and MALDİ-TOFF Mass spectra of compound 4d.


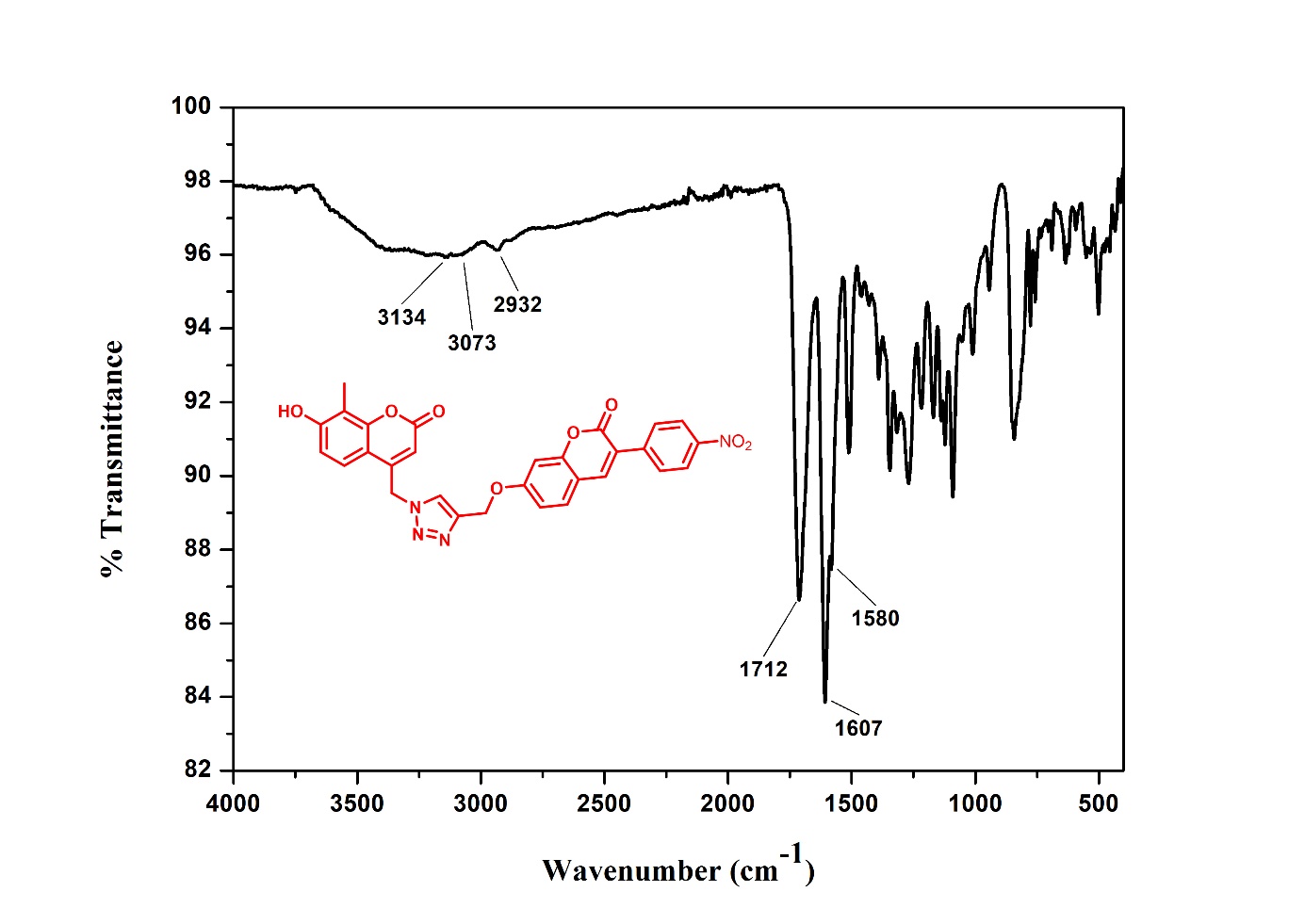


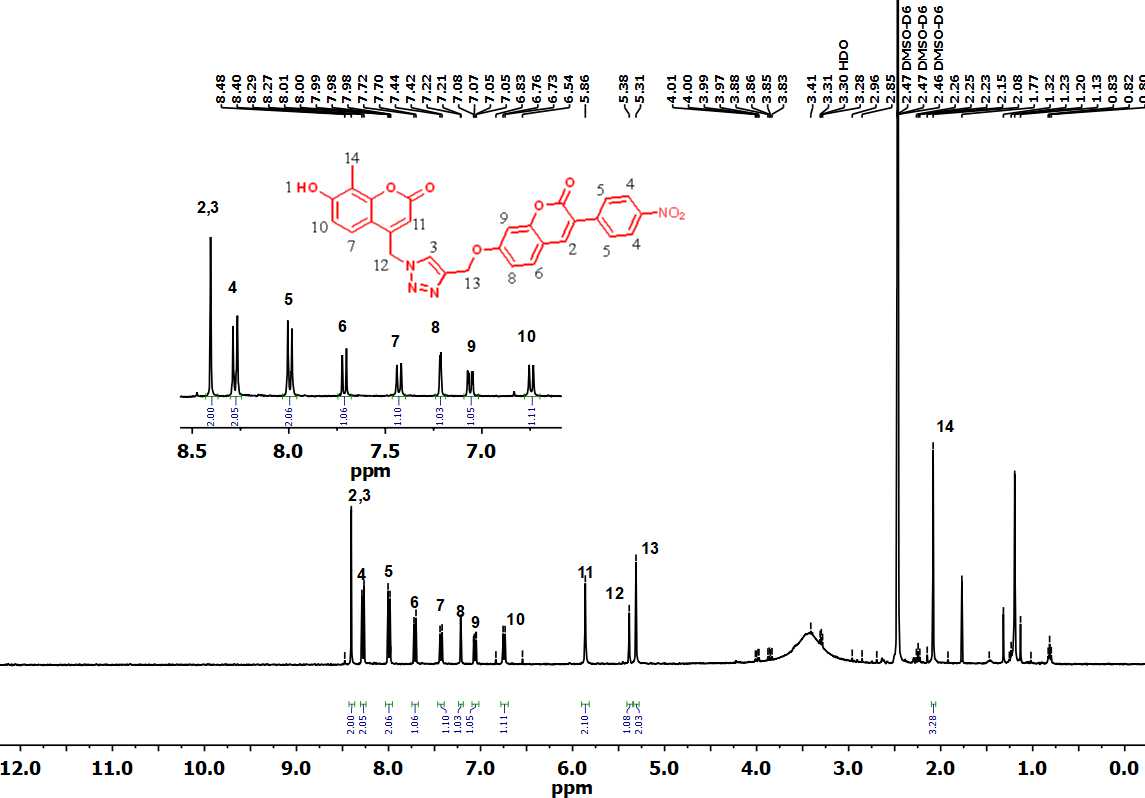


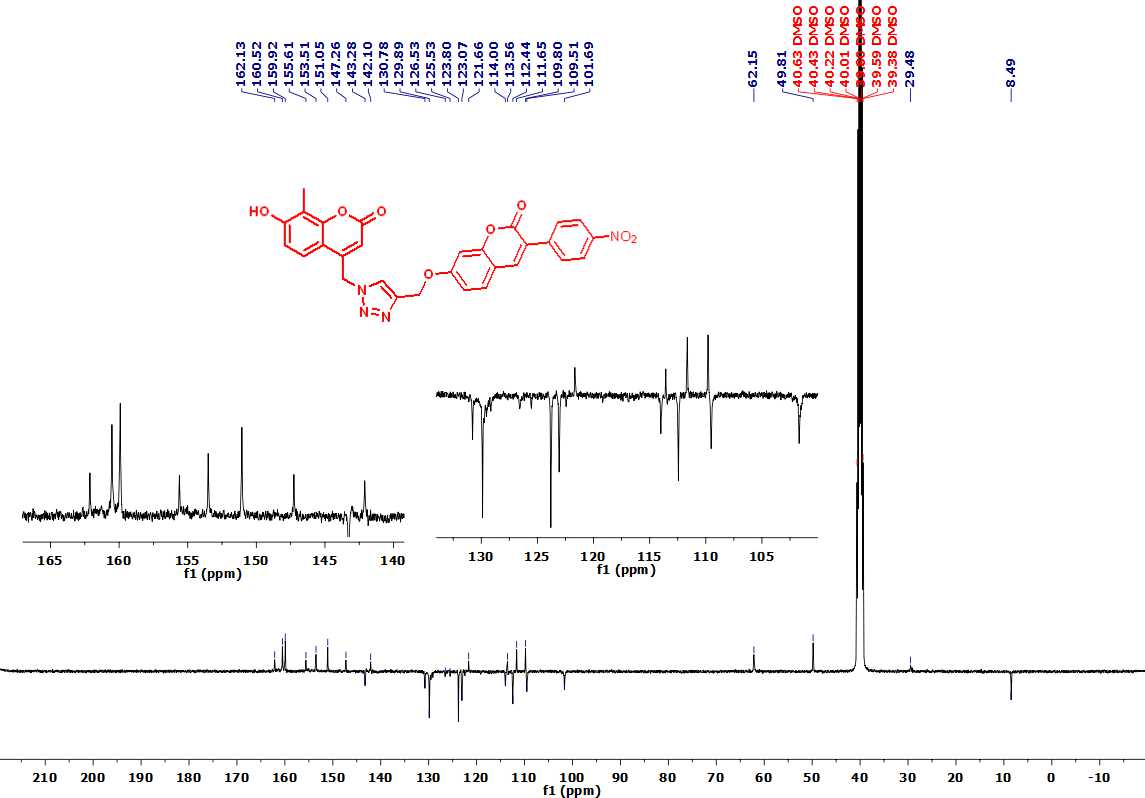


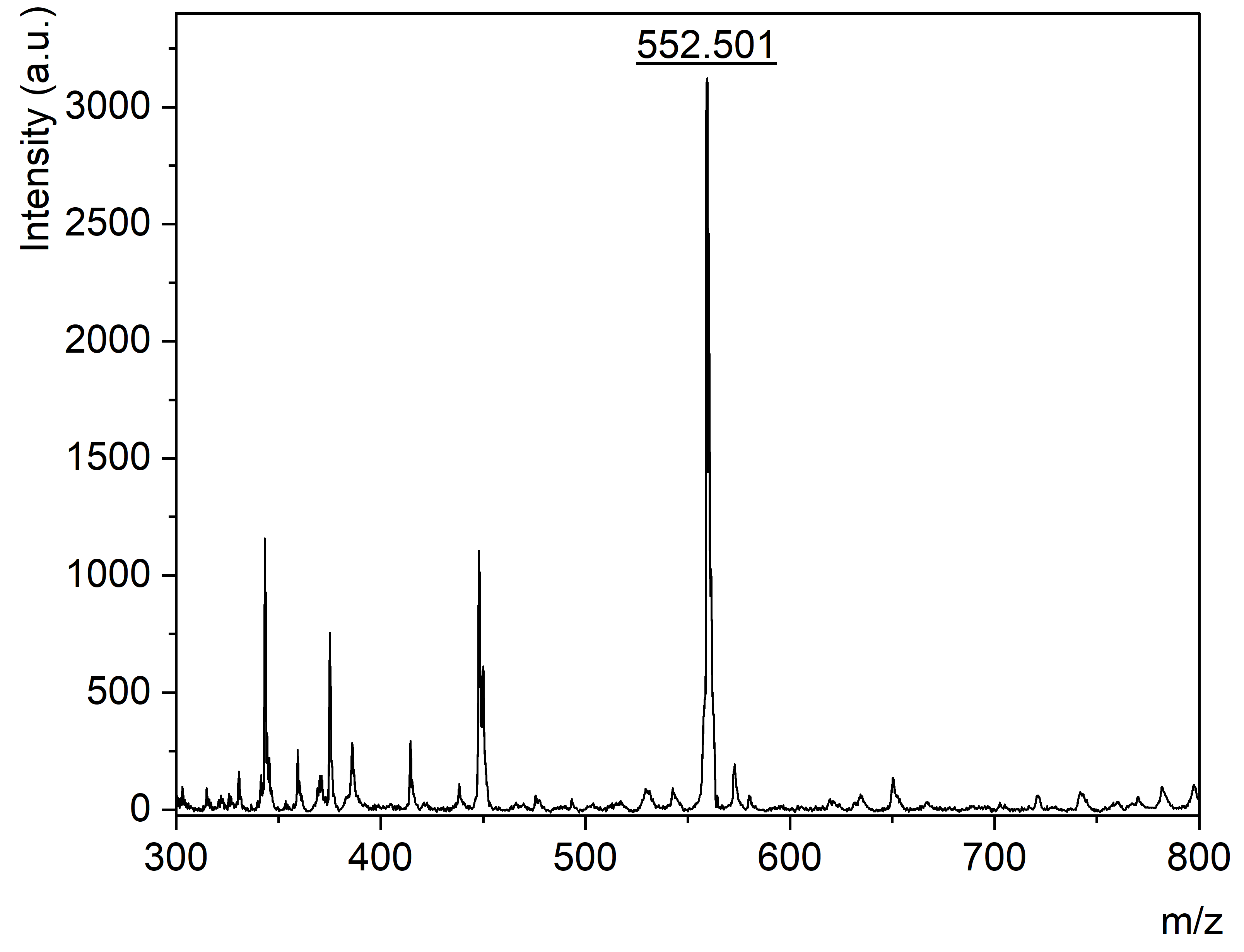


**Figure S6:** FT-IR, ^1^H and ^13^C NMR spectrum and MALDİ-TOFF Mass spectra of compound 4e.

| 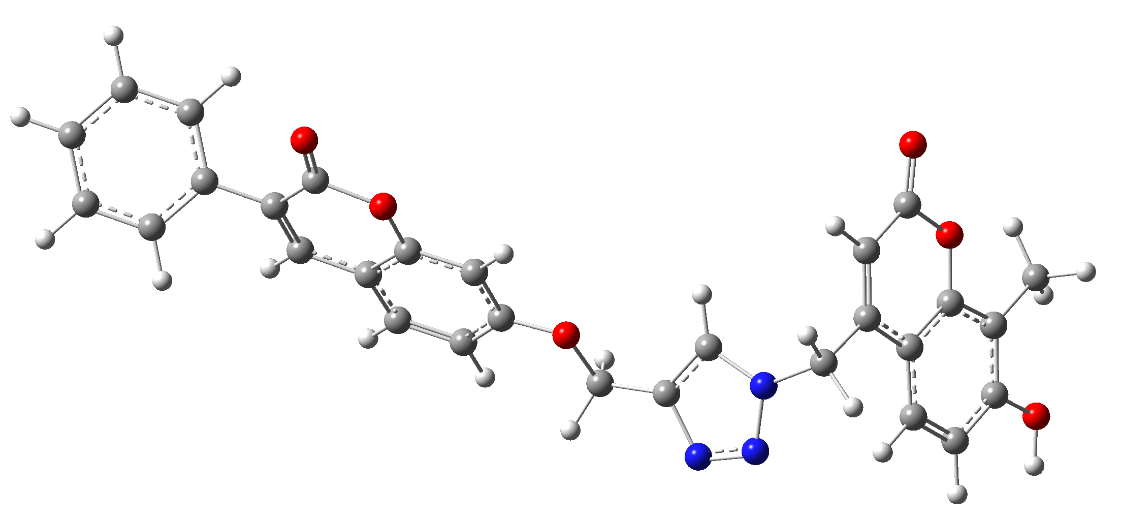 |
| --- |
| (a) |
| 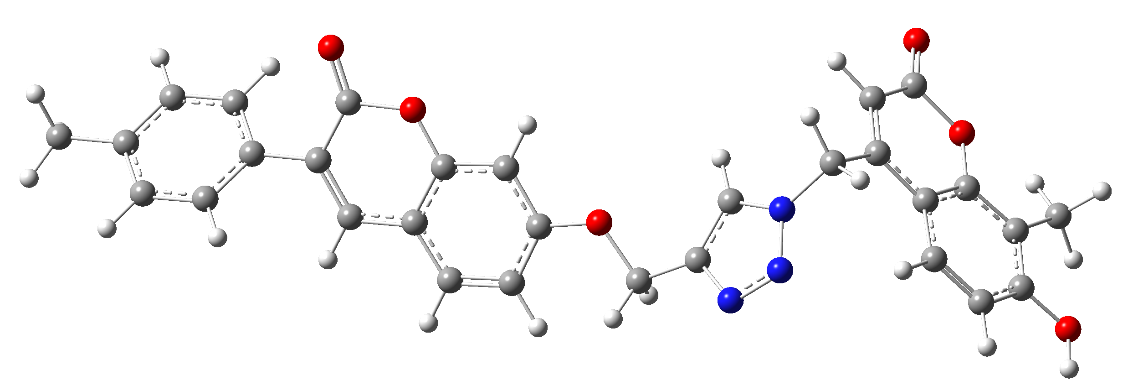 |
| (b) |
| 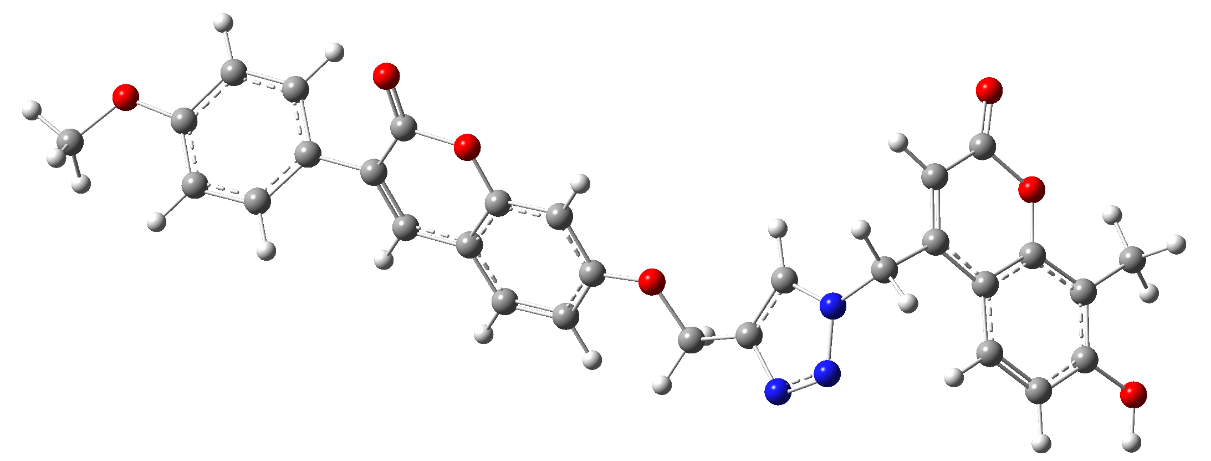 |
| (c) |
| 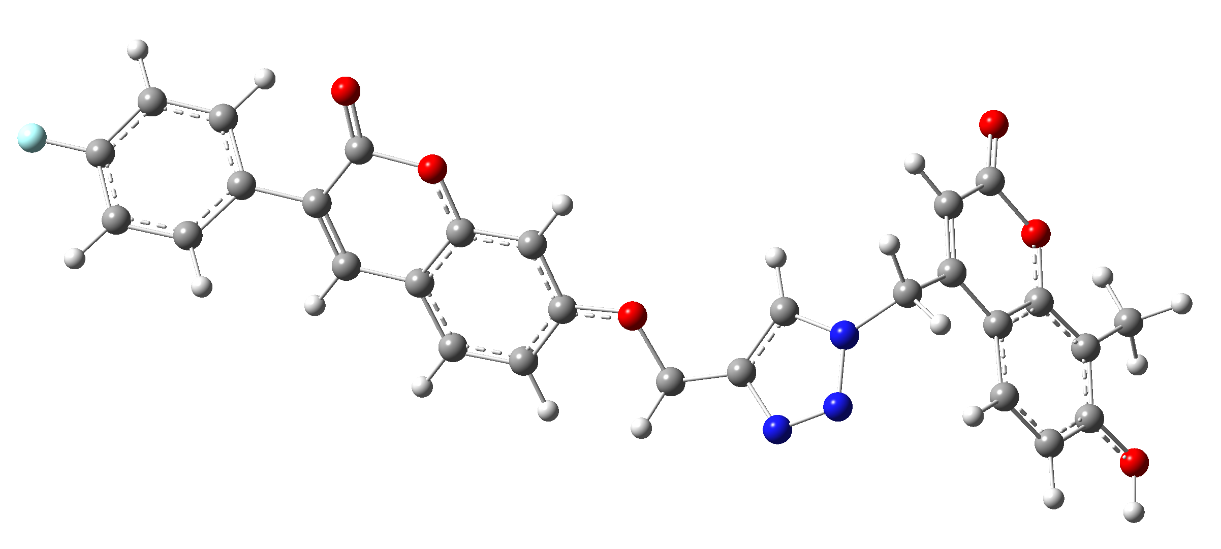 |
| (d) |
| 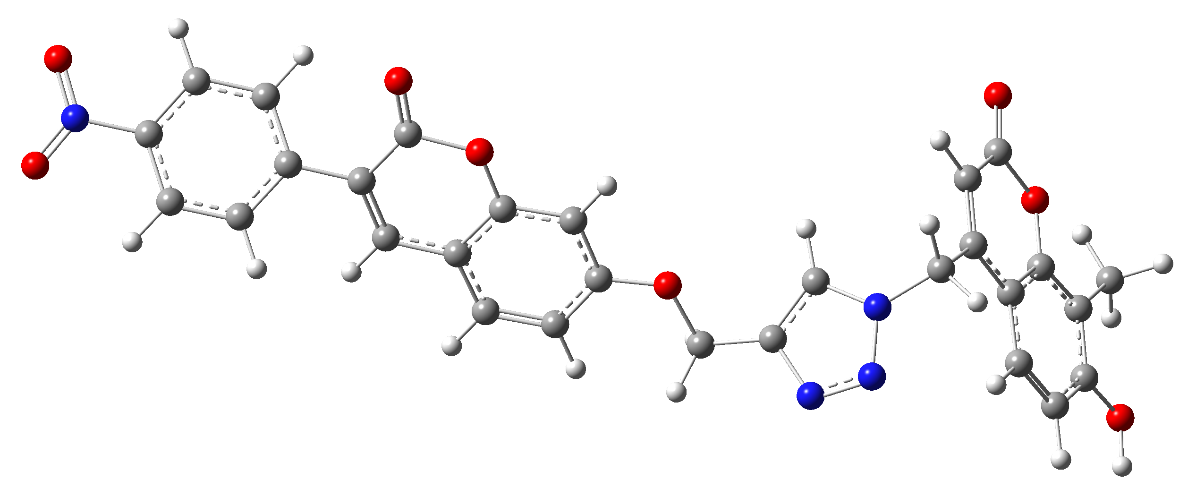 |
| (e) |

**Figure S7.** Optimized structures of compounds

| 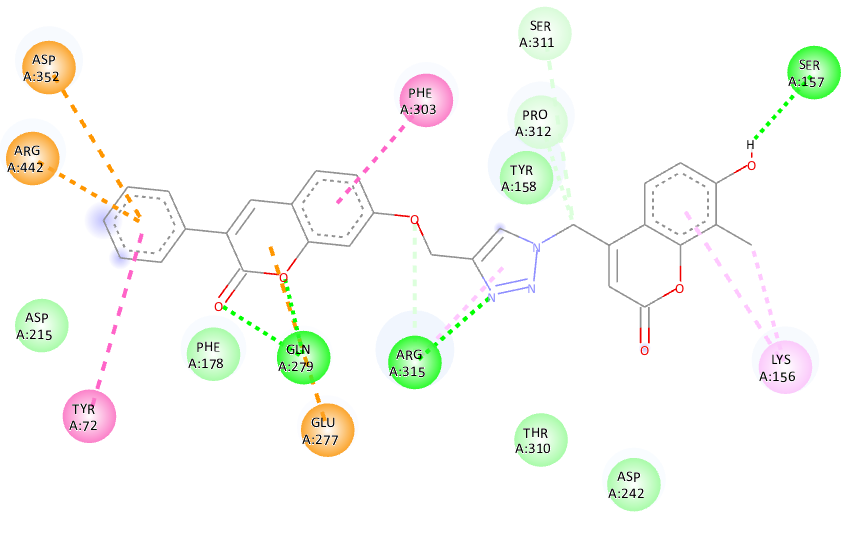 |
| --- |
| 4a |
| 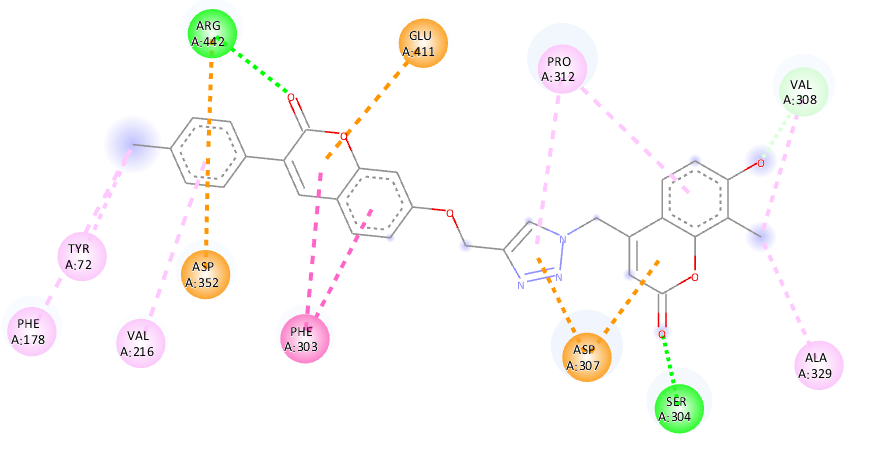 |
| 4b |
| 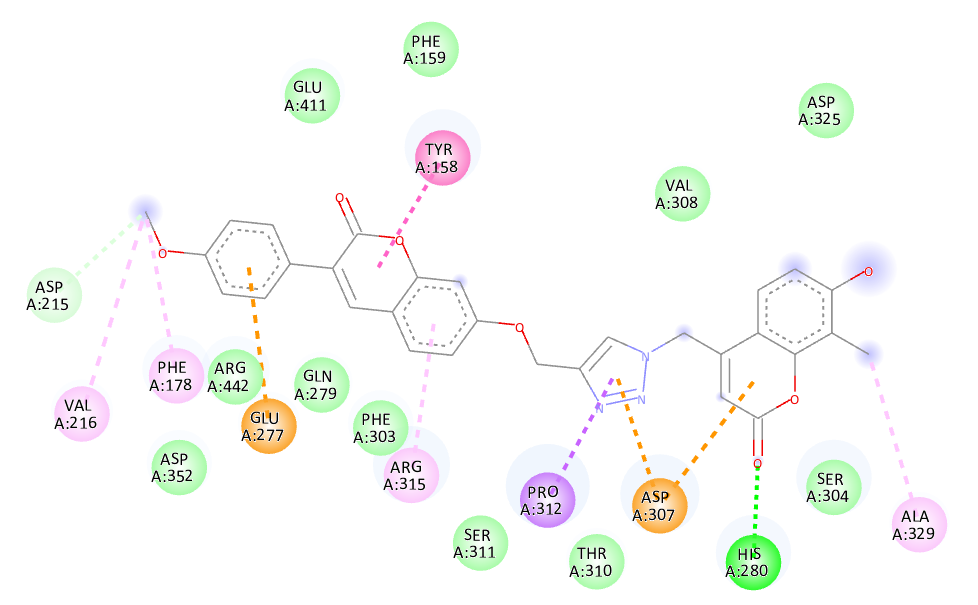 |
| 4c |
| 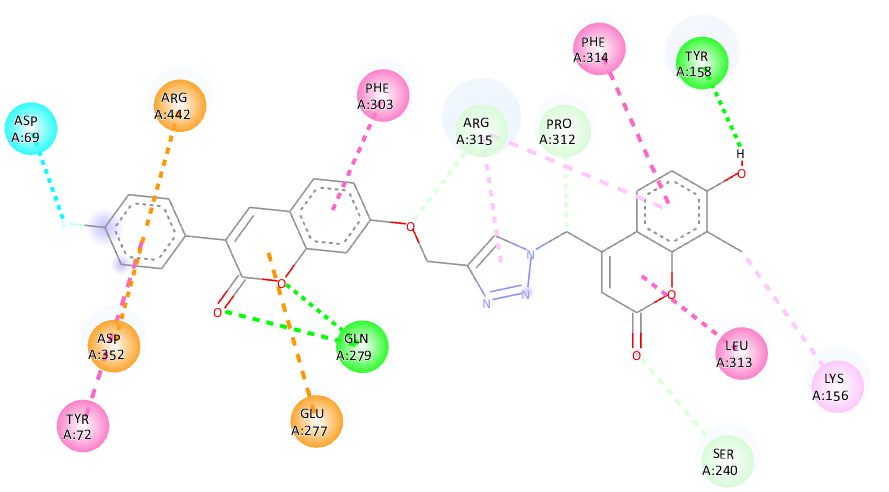 |
| 4d |
| 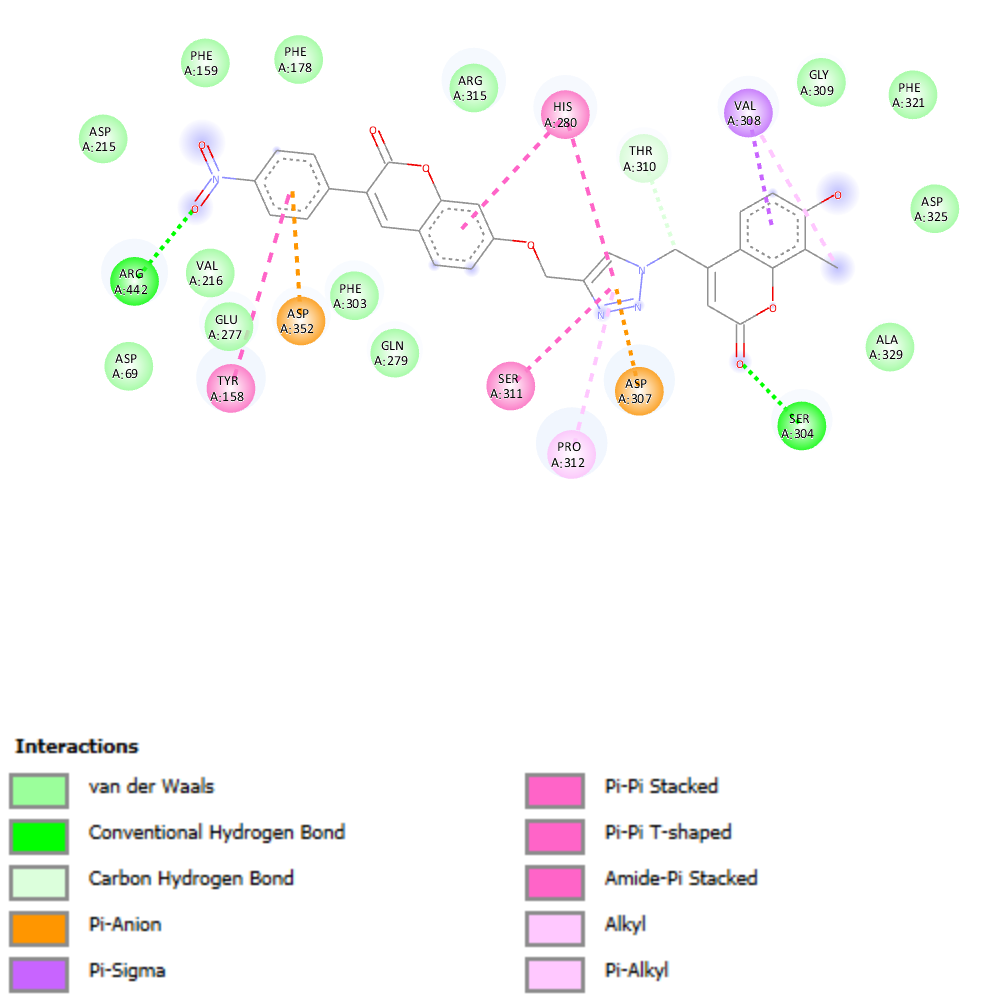 |
| 4e |

**Figure S8**: 2D view of α-glycosidase (PDB No: 3A4A) interactions with compounds.


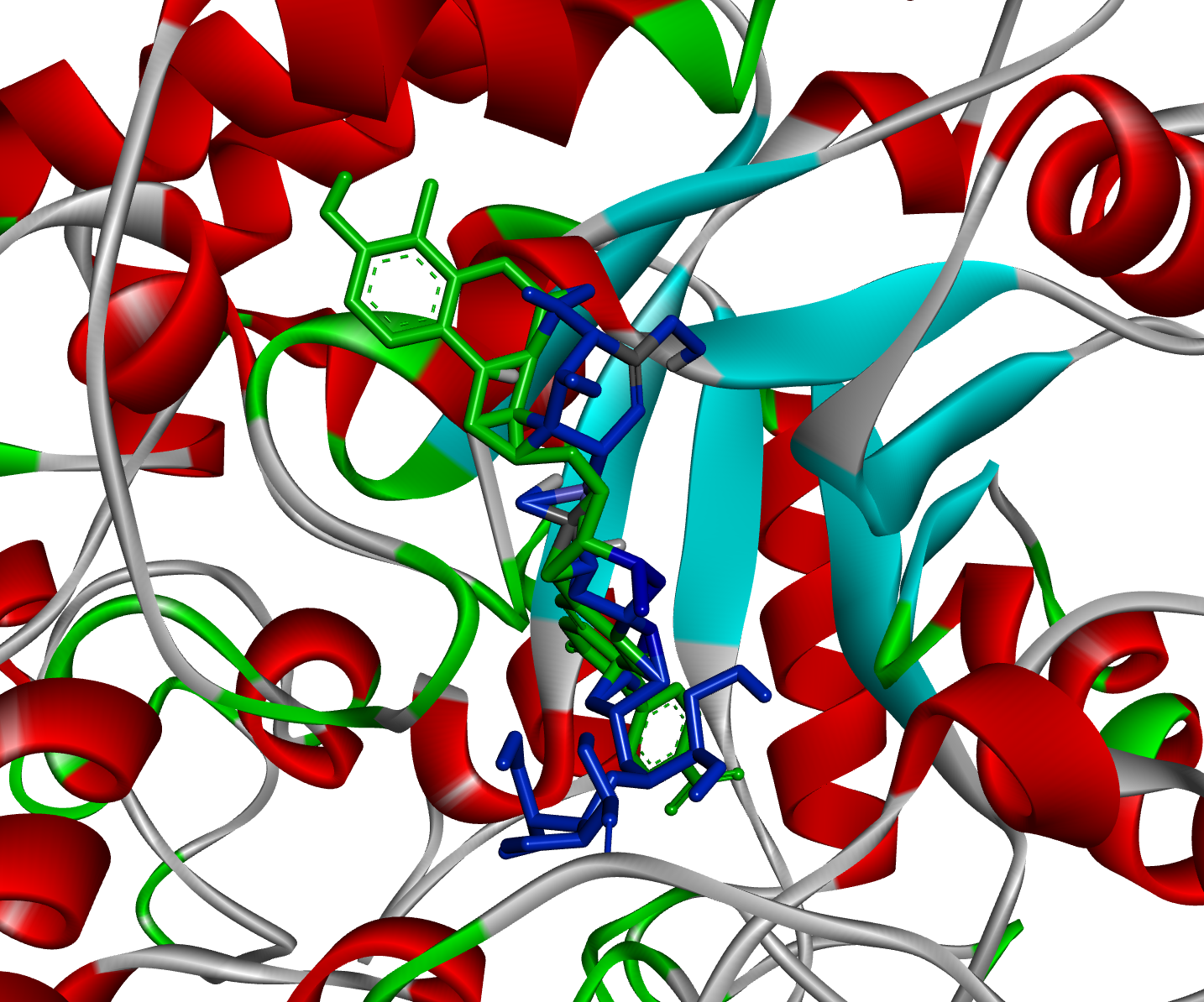


**Figure S9:** Acarbose (blue) and most potent compound 4e (green) superimposed in the active site pocket.

| 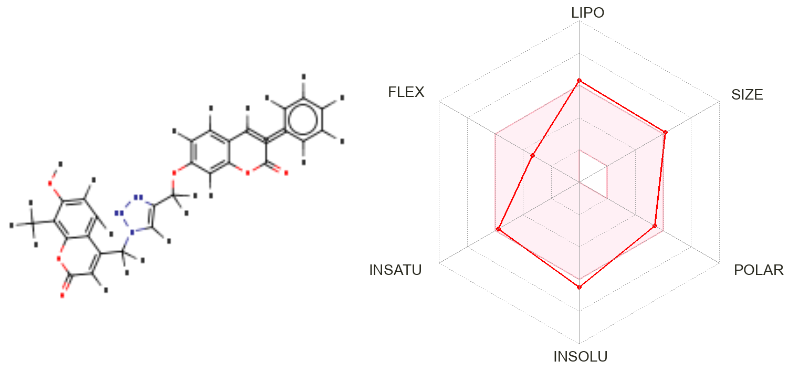 |
| --- |
| 4a |
| 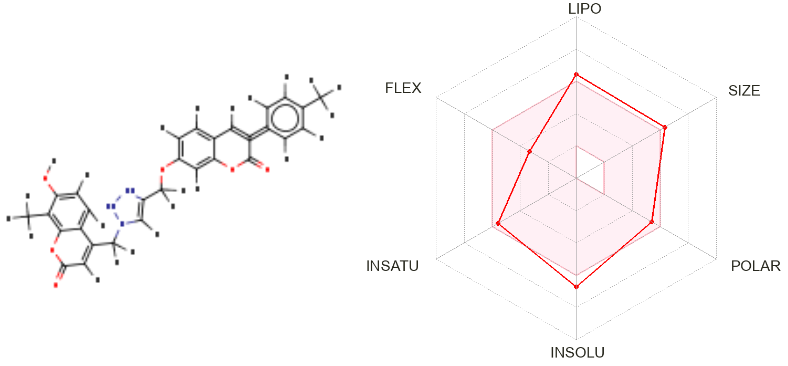 |
| 4b |
| 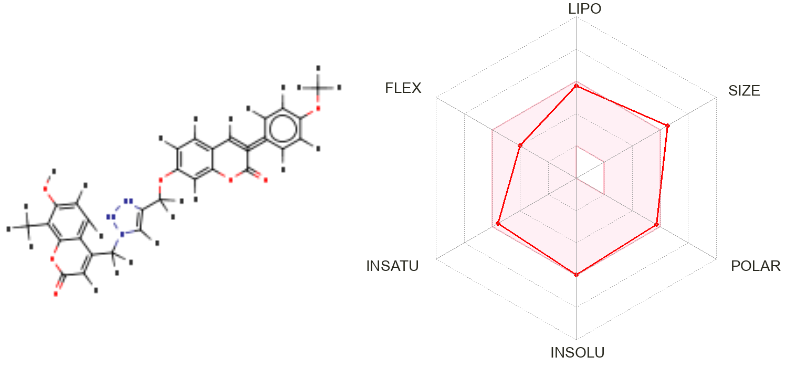 |
| 4c |
| 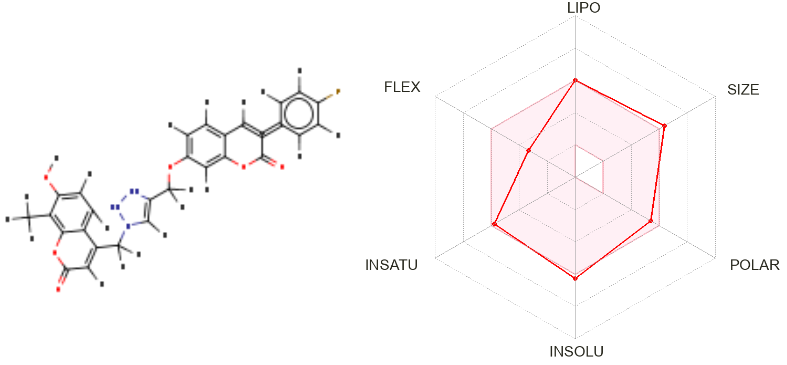 |
| 4d |
| 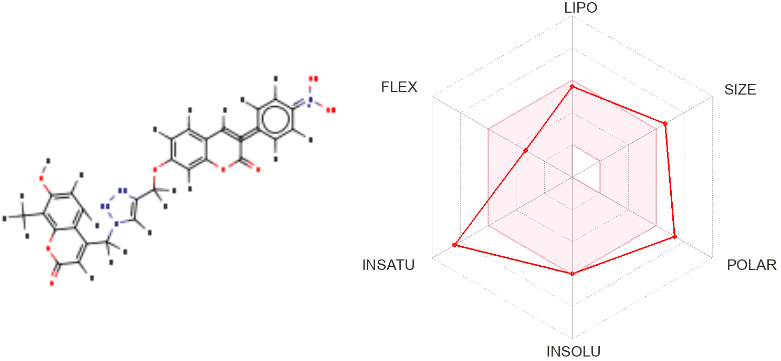 |
| 4e |

**Figure S10**: Color regions and physicochemical parameters of compounds.

**Table S1.** Some physicochemical properties such as HOMO, LUMO energies, dipol moment, and polarizability of the compounds.

|  | **E_HOMO_** | **E_LUMO_** | **DE** | **Dipol Moment** | **Polarizability** |
| --- | --- | --- | --- | --- | --- |
| **4a** | -5.87 | -2.01 | 3.86 | 7.63 | 347.42 |
| **4b** | -5.76 | -2.00 | 3.76 | 7.40 | 336.23 |
| **4c** | -5.78 | -2.10 | 3.68 | 6.82 | 321.28 |
| **4d** | -5.93 | -2.14 | 3.79 | 8.11 | 349.36 |
| **4e** | -6.20 | -2.43 | 3.77 | 9.03 | 363.68 |
